# Filling the human resource gap through public-private partnership: Can private, community-based skilled birth attendants improve maternal health service utilization and health outcomes in a remote region of Bangladesh?

**DOI:** 10.1371/journal.pone.0226923

**Published:** 2020-01-17

**Authors:** Jahangir Hossain, Anne Laterra, Rina Rani Paul, Ahsanul Islam, Faisal Ahmmed, Bidhan Krishna Sarker

**Affiliations:** 1 Health and Nutrition Team, CARE Bangladesh, Dhaka, Bangladesh; 2 Sexual Reproductive Health and Rights Team, CARE USA, Atlanta, Georgia, United States of America; 3 International Centre for Diaarrhoeal Disease Research, Dhaka, Bangladesh; Liverpool School of Tropical Medicine, UNITED KINGDOM

## Abstract

**Background:**

In Sunamganj there are fewer than four skilled providers per 10,000 population and just 27% of births are assisted by a skilled attendant. We evaluate a private community skilled birth attendant (P-CSBA) model, developed through the GSK-CARE Frontline Health Worker Programme, designed to address this gap and report on changes in service utilization and health outcomes from baseline to three years post-baseline.

**Methods:**

This analysis presents the results of a pre-post cross sectional design. A baseline survey (n = 1800) was conducted using a multistage cluster sampling approach. Three years post-baseline a second cross-sectional survey (n = 1755) was conducted across the same project area. To describe demographic characteristics of the study participants descriptive statistical techniques were used as appropriate. Logistic and multiple logistic regression, controlling for a comprehensive set of covariates, were used to assess odds ratios for key maternal health behaviors and outcomes.

**Results:**

Birth planning and the use of key maternal health services improved from baseline to follow-up. There was a dramatic increase in the proportion of respondents reporting skilled attendance at birth (aOR: 2.18, p = .001). Women also reported significantly fewer complications during the prenatal (aOR: .30, p<.001), labor and delivery (aOR: 0.41, p<.0001) and postnatal periods (aOR: 0.32, p<.0001).

**Conclusion:**

Private-sector approaches, when coupled with robust efforts to strengthen and collaborate with the public sector, can work successfully to deliver services in underserved communities. The success of this model lends credence to the growing appreciation that reaching our development targets will require governments to work in partnership with private sector actors and highlights the potential of private-public partnerships as we drive towards universal health coverage.

## Introduction

### Global context

After missing the mark in 2015 and failing to reduce the maternal mortality ratio (MMR) by three quarters [1], the global community came together around the Sustainable Development Goals and committed to refocus its efforts on a new global MMR target of less than 70 by 2030 [2]. It is widely acknowledged that the capacity of the global health workforce will need to be transformed in order to meet this and other SDG targets [3–5]. A sufficient concentration of qualified health workers is strongly correlated with key positive health outcomes, including maternal survival [6–9]. Unfortunately, a global shortage of providers and poor retention of existing workers continues to act as a critical barrier to the successful delivery of the health services needed to both reach universal health coverage and achieve the SDGs [10]. The scale of this shortage is staggering; a recent report estimates a global shortfall of more than 17 million health-care workers with South-East Asia requiring an additional seven million providers [4].

### Bangladesh context

The global and regional trends described above also exist in Bangladesh. Sunamganj District, a remote region characterized by wetlands and flooding, is within Sylhet Division where the MMR is more than double the national estimate at 425 deaths per 100,000 live births, the highest of any of the country’s eight divisions [1, 11]. Many of these deaths can be attributed to the high proportion of births outside of a health facility (65%) [12] and low proportion of births assisted by a skilled attendant (38%) [13].

While there are human resource shortages across Bangladesh, rural areas currently experience the largest gap [14]. In Sylhet, there are fewer than four skilled providers per 10,000 population, the lowest across Bangladesh and dramatically below both the WHO minimum standard of 23 and the SDG Index Threshold of 44.5 [4, 14, 15]. As this shortage of qualified health workers persists, many people are forced to seek health care from non-qualified providers in the informal sector [16–18]. Due to this dependency on private providers, out-of-pocket spending on health care services is high. In 2014, nearly 60% of Bangladesh’s total expenditures on health were out-of-pocket; clients often face no other option than to be burdened with high costs for poor quality services from unskilled providers [19].

The Government’s attempts to address this gap have been met with only limited success [11, 20]. In 2003 the Government of Bangladesh introduced a community skilled birth attendant (CSBA) training program designed to train and deploy two CSBAs in each union of the country. The CSBAs were most often selected from the existing public healthcare workforce. Evaluations of this program found that even though CSBA’s were more available in rural areas, utilization of their labor and delivery services was low. Seven years following the initiation of this program less than 1% of all births were attended by a CSBA [11]. Assessments found that CSBAs were often busy providing non-maternal health services that were part of their defined job responsibilities prior to training and so unavailable when women went into labor; they typically were not residents of the communities they served so could not be called upon in an emergency; they had inadequate monitoring and supervisory support; and communities lacked awareness about their services leading to mistrust and creating an unsupportive environment [21, 22].

In this environment, Traditional Birth Attendants (TBAs) remained a primary source of delivery care. In contrast to CSBAs, TBAs were able to provide round-the-clock care, perform house calls, and accept non-monetary payment, all advantages in these settings [23]. TBAs are usually a part of the communities in which they work and so are familiar to their clients, cementing a strong faith in their skills and experience [18, 24]. Religious beliefs and social norms also impede women from using facility-based providers for labor and delivery services. Muslim women feel pressure to maintain *purdah* and so are reluctant to be seen by a provider based at the health facility, and likely male, and instead seek home-based services from female TBAs [24].

In response to these complex challenges CARE, with funding from GSK’s Frontline Health Worker Programme, formed a public-private partnership with the Government of Bangladesh to design and implement the Private Community Health Worker Initiative in Sunamganj. In this article, we report on changes in service utilization indicators and health outcomes observed from baseline to three years post-baseline in the intervention area. Lessons learned through this analysis may inform decisions about potential expansion of this model to remaining unions of Sylhet and, indeed, to other regions or contexts facing similar poor maternal health outcomes due to human resource constraints and restrictive community practices and norms.

## Program description

Informed by lessons learned from similar efforts in other contexts including Indonesia’s village-based midwife program [25], the results of the evaluation of the Government’s CSBA program, and the findings of a participatory scoping exercise, CARE, the Ministry of Health and Family Welfare (MOHFW), and the Obstetrics and Gynecology Society co-designed an intervention to successfully address the human resource crisis and improve maternal health outcomes in 10 sub-districts and 50 unions of Sunamganj. Specific components of the intervention included:

- Selection and training of private- community skilled birth attendants (P-CSBA);
- Social entrepreneurship capacity building;
- Community engagement and mobilization to introduce P-CSBAs, set prices, and promote services;
- Formal partnership and linkages to local government to ensure quality and coordinated service provision; and
- Accountability mechanisms to mobilize resources.

### P-CSBA selection

P-CSBAs were recruited and selected through a consultative process with representatives from local government bodies, community groups and local health departments. Women who had at least ten years of schooling and who intended to live within their service areas were eligible to apply. The final cohort was selected based on the results of a written test and interview. The project prioritized selection of ever-married women between ages 25–40. In this context, ever-married women are less likely to migrate away from the service area, ensuring that investments made in training and development are well placed. This age range was prioritized because, in this region, younger women are often still in child-bearing years and pregnancy and child care often impedes ability and willingness to travel long distances and spend many hours away. As of January 2016, 401 applications were received, 247 were eligible and passed the exam, and 174 were selected to receive the first round of training. Among those selected for training, 12 discontinued and 7 were added from the waiting list. In total, 168 women completed the training.

### Competency-based skills training

Selected candidates completed a six-month, residential CSBA training and were thus accredited by the Bangladesh Nursing Council as skilled providers. Initial classroom training was followed by 3 months of field training and supplementary instruction in primary health care and community based integrated management of childhood illness.

To ensure that P-CSBAs had the skills and tools needed to sustainably deliver services, and ultimately become self-sustaining, they also participated in social entrepreneurship training. During this two-day training P-CSBAs explored and interpreted results from market research conducted to understand where community members were currently accessing care, developed individual business and marketing plans, and were linked with public and private pharmaceutical and commodity suppliers. This training also provided a space for P-CSBAs to plan how they would engage with TBAs to minimize unhealthy competition and encourage collaboration.

### Service delivery package

Each P-CSBA serves an area with a population of approximately 8,000 and is supported by ten community health workers, allocated from the public health system. Their service delivery package includes:

- antenatal care services;
  - Identification of pre-existing health conditions (weight, blood pressure, anemia and nutrition screenings)
  - Early detection of pregnancy-related complications (blood glucose test, urine testing)
  - Health promotion and disease prevention (nutritional counseling, micronutrient powders, iron and folic acid supplements, family planning counseling and tetanus vaccination evaluation and referrals)
  - Birth preparedness and complication planning
- assistance during normal, uncomplicated delivery;
- postnatal and newborn care services;
- community integrated management of childhood illnesses;
- referral to secondary services for complications during labor and delivery or for other services outside of their scope;
- family planning counseling;
- family planning method provision (contraceptive pill and condom) and referral; and
- sale or distribution of feminine hygiene products.

### Community engagement

#### Introduction and price setting

After training was complete, project staff and Union Parishard (local government) facilitated introductory meetings where P-CSBAs and their services were presented to the communities. Prices for their services were then set in a transparent, negotiated process with involvement and agreement between P-CSBAs, the Union Parishard and community leaders.

#### Community support system

Recognizing that previous efforts to establish a cadre of CSBAs were challenged by the lack of community awareness of and support for their services, CARE included its Community Support System (CmSS) approach in this model [26–28]. These Community Support Groups identify and track each pregnant woman, helping her and her family develop safe birthing plans and pool community funds to subsidize care to low-income households and support emergency transport. They also convene meetings with community members to review performance indicators and ensure accountability and commitment to improving maternal health outcomes. Community Support Groups play an active role in promoting P-CSBA’s services and supporting their integration and access to communities. In some cases, Community Support Group members accompany P-CSBAs when they are called away from their homes late at night to attend deliveries, making it easier for them to deliver their services.

### Community health workers

Each P-CSBA was linked to and supported by ten Community Health Workers. These volunteer health workers supported P-CSBAs by serving as a more direct link between community members and the P-CSBA; identifying, mapping and registering pregnant women; and socializing P-CSBA services and key health information and messages.

### Partnership with local government

In keeping with the public-private partnership model, a formal Memorandum of Understanding between local governments, CARE and the P-CSBA’s was established to link providers to referral health facilities and, in some cases, ensure that P-CSBAs were eligible for the reimbursement scheme funded through local government budgets. In this vein, CARE worked with local governments to estimate funds needed to support this work and ensure that specific budget line items were created and maintained for this purpose including financial resources to support transportation from communities to facilities in the case of emergencies. Data sharing between P-CSBAs and the public sector is also a crucial component of this relationship. These efforts ensure that public officials could represent not just the growth of service provision in the public sector but also include services provided by the P-CSBAs as part of the larger success story. This public-private partnership is particularly successful due to the unique nature of this region, specifically chronic shortages of public health care providers and failed efforts to recruit and retain them. In this context, local governments are especially cognizant of the complementary role that P-CSBAs play in filling a critical human resource gap and linking communities to public health care providers and facilities through close partnership with the public sector.

### Remuneration

When P-CSBAs register pregnant women in their communities they categorize households in terms of wealth quintile using a simple, five-question tool. Women from households categorized as extremely poor receive P-CSBA services free of charge and others are charged on a sliding scale. In some cases, local governments and/or community support groups subsidize the services provided to extreme poor households so that P-CSBAs can continue to provide these services.

### Monitoring, supervision and support

To ensure strong oversight of this new cadre, government service providers from health and family planning departments and nurse field trainers conducted quarterly monitoring visits. These visits served as a quality assurance mechanism and informed decisions around the focus of the sub-district skills labs where refresher trainings were provided. Special attention was paid to weak P-CSBAs identified through monthly performance reports and supervisory observations. These providers were linked to a mentoring support system and attached to local hospitals. As part of this public-private partnership, all P-CSBAs participated in monthly performance review meetings with public providers and local government at the sub-district level.

## Methods

### Study design

This study was reviewed and approved by the International Centre for Diarrhoeal Disease Research, Bangladesh’s research review committee and ethical review committee [PR#12083 and PR#15124]. All respondents provided written informed consent prior to the interview. Participants who were illiterate included their thumbprint accompanied by enumerator signature in lieu of their own. The study was a pre-post cross-sectional design. Prior to project implementation, a baseline survey (n = 1800) was conducted using a multistage cluster sampling approach. The sample size was calculated to detect a 30% change in the neonatal mortality rate in Sylhet from the prevailing rate prior to baseline [29]. Given the hypothesized effect size our power analysis determined a sample of 1800 women was needed (power = .845, α = .05, non-response = 10%, and design effect = 1.03). About three years post-baseline a second cross-sectional survey (n = 1755) was conducted across the same project area.

### Baseline survey

In November and December of 2013, a cross-sectional survey was conducted among 1800 women aged 15–49 years of age who had given birth within the previous 5 years (60 months). At first stage, one union was randomly selected from each of the 10 intervention sub-districts. A list of all 355 villages in these 10 unions was then compiled. Using UNICEF’s probability proportional to size method [30], 30 villages were selected from within the 10 sub-districts to serve as the primary sampling units.

The World Health Organization Expanded Program on Immunization (EPI) method was employed for household selection [31]. The midpoint of each of the study areas was determined in consultation with the community. From this midpoint, data collectors selected a start direction using a random spill of a bottle and visited every subsequent household. If an eligible woman living in a given household was reported as being absent then data collectors returned two more times in an attempt to complete the interview. If more than one eligible respondent was identified in a given household enumerators randomly chose the person to be interviewed. This process was repeated until 60 women were surveyed in each village. If enumerators were unable to identify 60 eligible women in the selected village they surveyed additional women in the next sampled village.

### Follow-up survey

From January to March 2016 a second cross-sectional survey was conducted among 1755 women aged 15–19 years of age who had given birth within the previous 1 year (12 months). The P-CSBA service area was used as the first stage cluster unit. In general, each union has 3 P-CSBAs although there were several larger unions where 4 P-CSBAs were deemed necessary due to their size. Several catchment areas where P-CSBAs dropped out or were not operational were excluded from the sampling frame. In total, 149 P-CSBA catchment areas were included in the initial sampling frame corresponding to the 10 intervention sub-districts used as the baseline sampling frame. At the first stage, 35 PCSBA service areas were randomly selected. To select the primary sampling unit, a list of all villages in the 35 selected service areas was then compiled and one village was randomly selected using simple random sampling technique from within each of the service areas for a total of 35 villages. Household data collection preceded in the manner described above.

### Survey design and data collection

Semi-structured questionnaires (S1 and S2 Files) were developed based on the research questions and objectives of the study. Once developed, all data collection tools were translated to Bangla, back-translated and pre-tested to ensure validity. Data collection was conducted by an experienced team of enumerators from the International Center for Diarrheal Disease Research (icddr,b) who were trained on the objectives of the study, format of the survey tool, and sampling procedures.

### Bias

At follow-up, service areas assigned to P-CSBAs that dropped-out of the intervention were excluded from the sampling frame. As a result, the analysis presented below applies to those fully-exposed to the intervention and not an intention-to-treat approach. Those P-CSBAs that dropped out of the intervention may not have done so randomly, their service areas could tend to be better or poorer performing, making it more difficult to compare baseline results which are representative of the entire intervention area and follow-up results which exclude those areas where P-CSBAs were not successful.

### Measures

#### Outcomes

We evaluated outcomes related to birth preparedness planning and service utilization related to the last pregnancy. These included: preparedness planning at last pregnancy, receipt of any antenatal care (ANC) from a skilled provider, receipt of sufficient ANC (at least 4 ANC visits) from a skilled provider, skilled attendance at birth, facility delivery, receipt of PNC from a skilled provider within 24 hours of birth, receipt of postnatal care (PNC) from skilled provider within 2 days after birth, self-reported prenatal complications, self-reported labor and delivery complications, and self-reported postnatal complications.

Skilled providers were defined in accordance with the DHS definition and included qualified doctor, nurse, midwife, family welfare visitor, and P-CSBA. For self-reported complications women were asked and prompted about their experience with the following: Excessive bleeding or hemorrhaging, convulsions, severe headaches, severe anemia, blurred vision, edema, high blood pressure, elevated fever, unusual vaginal discharge, reduced fetal movement, prolonged labor (>12 hours), uterine prolapse, retained placenta, inverted nipples, tetanus, abdominal pain, and engorged breasts.

#### Covariates

We controlled for maternal age, maternal education, para, maternal religion, husband’s occupation (their primary occupation in the last six months), and wealth index in all models. The wealth index was constructed using a subset of indicators (durable goods, housing facilities, and housing materials) drawn from the Demographic and Health Surveys methodology [32].

### Analysis

For this analysis, to ensure comparability between samples taken at baseline and follow-up, the baseline sample was restricted to include only those respondents who had given birth within the previous 1 year. Data were managed using ORACLE and were exported to STATA 13.0 (Stata Corp LP, College Station, TX, USA) for analysis. To describe demographic characteristics of the study participants, frequencies, percentages, mean/median and standard deviations were used as appropriate. Student t-tests for continuous normally distributed variables and chi-squared test for categorical variables were used to compare the changes of women’s demographic characteristics and service utilization between baseline and follow-up. We performed logistic regression to assess crude odds ratio (OR) for outcome variables described above followed by multiple logistic regression, controlling for covariates described above. Estimation of standard errors takes into account clustering at the level of the primary sampling unit.

## Results

### Characteristics of study sample

The baseline and follow-up samples differed significantly across most observable characteristics apart from mean age (Table 1). On average, women at baseline reported greater parity than those at midline (3.14 vs. 2.83). This change was statistically significant (p = .002). Distribution of educational achievement differed significantly between baseline and follow-up (p<.001). Fewer respondents reporting having received no education while all levels of formal education increased. This change is consistent with national trends over the study period where the proportion of women having never attended school fell from 34% in 2007 to 24% in 2014 [33]. Similar trends have been observed in Sylhet Division. Likewise, the distribution of husband’s reported occupation varied between baseline and follow-up (p = 0.008). With an increase in those reporting work in the service industry or overseas, and in business and all other categories experiencing a decrease. A significant decrease in the proportion of respondents self-identifying as Hindu was observed, from 22% at baseline to 9% at follow-up (p<.001). This change is in line with changes seen at the national level where the proportion of the population identifying as Hindu decreased from 9.5% to 8.3% from 2011 to 2014, [11, 12] and may be explained in part by an increase in violent incidents targeting religious minorities, of which Hindus are a member, which began around 2013 [34]. Both the wealth index and distribution of wealth differed significantly between baseline and follow-up samples. The follow-up sample was less likely to include respondents from the first (poorest) and second quintiles and more likely to include respondents from the third, fourth and fifth quintiles. To ensure our analysis controlled for these differences between baseline and follow-up samples all variables in Table 1 were included as covariates in the multivariate regression analysis.

**Table 1:** Characteristics of study samples at baseline (2013) and follow-up (2016).

Characteristics	Baseline (n = 479)	Midline (n = 1755)	p-value
**Mean of age (SD) in years** ^a^	25.91 (5.36)	25.44 (5.37)	0.092
**Para**^a^**, mean (SD)**	3.14 (2.02)	2.83 (1.82)	0.002***
**Education, % distribution** ^b^			
No formal education	40.29	25.41	
Primary (1–5 Class)	38.00	47.46	
Junior secondary (6–8 class)	11.69	15.21	
Secondary & higher (9+ Class)	10.02	11.91	<0.001***
**Husband’s Occupation, % distribution** ^b^			
Service/Oversees worker	8.77	11.79	
Day laborer/rickshaw/van puller/transport worker	39.04	36.13	
Business	14.20	18.29	
Farming / Fishing/skilled labor	31.94	30.31	
Other	6.05	3.48	0.008***
**Religion, % distribution** ^b^			
Muslim	78.91	91.45	
Hindu	21.09	8.55	<0.000***
**Wealth index score** ^c^**, mean (SD)**	1.58 (0.84)	2.02 (0.76)	<0.000***
**Wealth quintile, % distribution** ^b^			
First (poorest)	28.60	10.31	
Second	21.29	18.40	
Third	18.58	18.97	
Fourth	17.12	26.27	
Fifth (wealthiest)	14.41	26.04	<0.000***

SD, standard deviation.

***P <.01,

^a^ P value from t test

^b^ P value from chi-square test

^c^ Wealth index score ranges: (Wealth index (WI) score range depends on which method we applied to generate the score, (If we applied principle component method, then WI score variable approximately follows standard normal variable with mean zero and variance one. So the range of WI score may lies between (-∞ to ∞), but it is ensure that 99.97% observations lies between (-2.0 to 2.0).

### Service utilization

#### Antenatal care

Table 2 compares the service provider distribution for ANC, labor and deliver, and PNC services between baseline and follow-up. Among those respondents who had accessed ANC services at baseline, more than half received this service from a Doctor (57%). Less than one quarter (17%) received services from a nurse, midwife or Female Welfare Visitor. Notably, unqualified providers such as pharmacists, drug sellers, and other untrained providers made up a relatively high proportion of service delivery (8.5% and 14% respectively). At follow-up, P-CSBAs delivered a greater proportion of ANC services (25.2%) than at baseline (3.5%). All other service providers, apart from CHCP / SACMO/ MA, experienced a decline in their relative proportions of ANC service delivery.

**Table 2:** Comparison of service provider distribution for ANC, birth attendance, and PNC services from baseline (2013) to follow-up (2016).

Maternal Health Service		
**Type of ANC provider** ^a^	**Baseline (n = 318)**	**Follow-up (n = 1436)**
Doctor	181 (56.9)	714 (49.7)
Nurse/ Midwife/ FWV	54 (17.0)	111 (7.7)
Govt. (public) CSBA	11 (3.5)	0 (0.0)
P-CSBA	0 (0.0)	362 (25.2)
CHCP/SACMO/MA	0 (0.0)	65 (4.5)
Pharmacist/ Drug Seller/ Village Doctor	27 (8.5)	32 (2.2)
Other	45 (14.1)	152 (10.6)
**Type of childbirth attendant** ^a^	**Baseline (n = 479)**	**Follow-up (n = 1755)**
Doctor	50 (10.4)	196 (11.2)
Nurse/ Midwife/ FWV	22 (4.6)	156 (8.9)
Govt. (public) CSBA	0 (0.0)	0 (0.0)
P-CSBA	1 (0.2)	171 (9.7)
CHCP/SACMO/MA	0 (0.0)	0 (0.0)
Pharmacist / Drug Seller/ Village Doctor	3 (0.6)	2 (0.1)
TBA	358 (74.7)	1030 (58.7)
Other	45 (9.4)	220 (11.4)
**Type of PNC provider (for first PNC check)** ^a^	**Baseline (n = 201)**	**Follow-up (n = 685)**
Doctor	75 (37.3)	277 (40.4)
Nurse/ Midwife / FWV	22 (10.9)	92 (13.4)
Govt. (public) CSBA	0 (0.0)	0 (0.0)
P-CSBA	0 (0.0)	107 (15.6)
CHCP/ SACMO/ MA	4 (2.0)	10 (1.5)
Pharmacist / Drug Seller/ Village Doctor	89 (44.3)	116 (16.9)
TBA	1 (0.50)	10 (1.5)
Other	10 (5.0)	73 (10.7)

^a^ If more than one source of care was mentioned, only the provider with the highest qualifications is considered in this tabulation

FWV, family welfare visitor; CSBA, community skilled birth assistant; P-CSBA, private community-based skilled birth attendant; CHCP, community health care provider; SACMO, sub-assistant community medical officer; MA, medical assistant.

#### Birth attendance

At baseline, among women who reported assistance at their most recent birth, TBAs were by far the most common source of assistance (75%). This reliance on TBAs, appears to have fallen over the course of the project to 59% of births at follow-up. The use of most other providers including doctors, nurses/ midwives/ and FWVs, and P-CSBAs all increased during the three years post baseline. Most notably, the use of P-CSBAs increased from 0.2% at baseline to 9.7% at follow-up.

#### Postnatal care

The changes in service provider distribution for PNC services are similar to those described above for labor and delivery services. At baseline, nearly half of all PNC recipients, received service from an unqualified pharmacist, drug seller or village doctor (44.3%). This proportion fell dramatically at follow-up where only 17% or PNC-users received care from these providers. At follow-up, PNC-users were more likely to have accessed services from doctors, nurses / midwives/ FWVs, or P-CSBAs than at baseline.

### Maternal and child health practices

Table 3 illustrates changes in birth preparedness planning activities, maternal health service utilization, and prevalence of self-reported complications between baseline and follow-up. Each row shows the results of a separate regression. The first, Panel A, without covariates, and the second, Panel B, with covariates.

**Table 3:** Changes in MNCH coverage from baseline (2013) to follow-up (2016).

	(A) Bivariate Regression Results		(B) Multivariate Regression Results	
Outcome	Crude OR^a^ (CI)	P Value ^c^	Adj. OR^b^ (CI)	P Value ^c^
Preparedness planning at last pregnancy				
Decided about place of delivery	2.40 (1.59–3.64)	0.000***	2.18 (1.42–3.33)	0.000***
Saved money	1.90 (1.43–2.51)	0.000***	1.74 (1.31–2.32)	0.000***
Prepared emergency transport	1.58 (0.98–2.53)	0.061*	1.44 (0.83–2.50)	0.195
Collected safe delivery kit	3.79 (1.85–7.76)	0.000***	3.57 (1.75–7.25)	0.000***
Women who took at least 2 of the initiatives as part of birth preparedness	2.64 (1.90–3.66)	0.000***	2.37 (1.66–3.38)	0.000***
Women who took at least 3 of the initiatives as part of birth preparedness	5.06 (2.67–9.57)	0.000***	4.90 (2.51–9.57)	0.000***
Received ≥1 ANC check from skilled provider at last pregnancy	1.94 (1.29–2.92)	0.001***	1.74 (1.15–2.63)	0.009***
Received ≥4 ANC checks from skilled provider at last pregnancy	1.55 (1.02–2.35)	0.039**	1.45 (0.98–2.16)	0.06
Skilled attendance at last birth (includes institutional deliveries and home deliveries with skilled health personnel)	2.36 (1.52–3.67)	0.000***	2.18 (1.40–3.42)	0.001***
Facility delivery at last birth	1.59 (0.97–2.63)	0.065*	1.50 (0.90–2.50)	0.115
Received PNC check from skilled provider at last birth	1.49 (1.01–2.19)	0.044	1.21 (0.82–1.77)	0.330
Received PNC check from skilled provider within 24 hours of last birth	1.98 (1.25–3.13)	0.003***	1.65 (1.06–2.56)	0.026**
Prenatal complications at last pregnancy	0.31 (0.25–0.40)	0.000***	0.32 (0.25–0.41)	0.000***
Labor and delivery complications at last birth	0.43 (0.32–0.57)	0.000***	0.41 (0.31–0.55)	0.000***
Postnatal complications at last birth	0.32 (0.25–0.40)	0.000***	0.32 (0.26–0.40)	0.000***

***p <.01,

**p<.05,

*p<.10

^a^ Crude odds ratios calculated by simple logistic regressions

^b^ Adjusted odds ratios calculated by multiple logistic regressions. Where all multiple logistic regression adjusted by covariates included are maternal characteristics (age, education, para, religion) and household characteristics (husband’s occupation, Wealth quintile)

^c^ All the p-value and confidence interval corresponding of all odds ratio are adjusted by survey cluster

#### Birth preparedness practices

Results showed large, positive, and statistically significant increases in 3 of the 4 birth preparedness planning practices at last pregnancy between baseline and follow-up, both with and without covariates. At follow-up, women were more likely to have discussed and decided about place of the delivery (aOR: 2.18, p<.001), saved money for birth related costs (aOR: 1.74, p<.001), and collected a safe delivery kit (aOR: 3.57, p<.001) than when compared to baseline. Women at follow-up were also 44% more likely to have organized emergency transport prior to delivery compared to those at baseline although this change was not statistically significant without or with covariate adjustment. Overall, when considered as a package, women at follow-up were more than twice as likely to have completed 2 of the 3 birth preparedness activities and nearly five times as likely to have completed 3 of the 4 birth preparedness activities than those at baseline (aOR: 4.90, p<.001).

#### Receipt of maternal, neonatal, and child health services

Self-reported receipt of antenatal, skilled labor and delivery, and postnatal care increased from baseline to follow-up. The odds of receipt of at least one ANC visit from a skilled provider were 74% higher among women surveyed at follow-up than those at baseline (aOR: 1.74, p = .009). The odds of receipt of the recommended 4 or more ANC visits (at time of study) also increased from baseline to endline although this change was only marginally statistically significant (aOR: 1.45, p = 0.063). At follow-up women were twice as likely to have delivered their most recent child with a skilled birth attendant than those at baseline (aOR: 2.18, p = .001). Likewise, odds of facility delivery increased from baseline to midline (aOR: 1.50, p = .115) although these changes were not statistically significant. Finally, receipt of postnatal care increased during the first three years of the project. At follow-up, a woman’s odds of having received a postnatal check from a skilled provider rose, although not statistically significantly, by 21%, when compared to baseline (aOR: 1.21, p = .330). A larger, statistically significant, change was observed for receipt of a postnatal check within 24 hours of delivery; odds were 65% higher than at baseline (aOR:1.65, p = .026).

#### Complications

We also analyzed self-reported incidence of complications before, during and after most recent birth. The odds of self-reported prenatal complications, hemorrhage, convulsions, severe headache, high fever, blurred vision, edema, high blood pressure, reduced / absent fetal movement, unusual vaginal discharge, severe anemia, and premature rupture of membrane (<37 weeks), were 70% lower at follow-up than among the baseline sample (aOR: .30, p<.001). The odds of self-reported complications during labor and delivery which included self-reported experience with hemorrhage, convulsions, high fever, unusual vaginal discharge, severe chest pain and rapid breath, prolapse / breach presentation, and prolonged labor (>12 hours), also fell by 59% during the project period (aOR: 0.41, p<.0001). Finally, analysis indicated a reduction in self-reported postnatal complications. When compared to baseline, women at follow-up were 68% less likely to have reported experiencing postnatal complications (aOR: 0.32, p<.0001). All changes in self-report of complications were statistically significant.

## Discussion

### Key results

Comparing data collected at baseline and three years post-baseline, this analysis demonstrates the feasibility of providing reliable, home-based, maternal health services in a remote and underserved area of Bangladesh through a robust public-private partnership model that links a cadre of private, community-based skilled birth attendants with communities and the public health system. It also demonstrates that building a strong, community-based cadre, of providers may not only increase community-based skilled birth attendance but also encourage women to seek out other sources of skilled care.

After training and deployment of this cadre with strong monitoring, supervision and support from public sector providers, birth planning practices and use of key maternal health services improved. Of particular note is the dramatic increase in the proportion of respondents reporting skilled attendance at birth over the course of this initiative. Over less than three years, women in the intervention area experienced more than a two-fold increase in skilled birth attendance. In comparison to recent national and regional trends this is impressive. For example, from 2014 to 2017 skilled attendance at birth nationally increased by only 18.29% (from 42.1% to 49.8% respectively). Regionally, in Sylhet, the increase was greater, 40.59% (from 27.1% to 38.1%) but not nearly as great as the increase experienced over the course of this study period [12, 35]. When comparing with similar interventions in other contexts, such as Indonesia’s “a midwife in every village” strategy, our results remain meaningful. In Indonesia this well-resourced effort produced, on average, an annual 7% increase in skilled birth attendance comparatively lower than what was observed in our data [36].

A comparison of providers of skilled attendance services indicates that these changes were driven not just by increases in the proportion of births attended by P-CSBAs but also by increases in those births attended by other skilled providers such as doctors, nurse, and midwives. This may be because P-CSBAs were well linked to these providers and so were able to confidently refer women for care. While the increase in the likelihood of delivering within a facility was not significant this may be because factors beyond the control of this program, namely long distances and flooding, continue to act as barriers to facility access.

Women also reported increases in receipt of postnatal care services from a skilled provider. Indeed, the proportion of PNC services rose for doctors, nurses, midwives and female welfare volunteers, and P-CSBAs. Surprisingly, we see a decline of ANC services provided by health facility-based staff (doctors, nurses, midwives and FWVs) from baseline to follow-up but an increase in services provided by P-CSBAs and CHCP/SACMO/MA providers suggesting that women may have switched from one skilled provider to another. Our qualitative monitoring data supports this. Women preferred the convenience, familiarity and low travel times and travel costs that the use of P-CSBAs and other community-based providers afforded. Women at follow-up also reported significantly fewer complications during the prenatal, labor and delivery and postnatal periods.

Mumtaz et al., in evaluating a similar private-sector community midwife (CMW) program in Pakistan, found that use of CMWs varied significantly based on contextual factors and that these factors were more important predictors of use of CMWs than individual factors, such as income level or education status, typically thought to dictate care-seeking behavior [37]. It’s possible that the Sunamganj context, a remote region with extremely challenging transportation barriers, and high out-of-pocket health expenditures for unskilled services, presented just the right contextual characteristics for this public-private partnership model to succeed. Further analysis of the Pakistani model, concluded that successful CMWs had an intrinsic sense of professionalism and strong business skills [38]. CARE’s adapted P-CSBA model, in recognizing the importance of these skills in addition to clinical expertise, provided social entrepreneurship training as part of the P-CSBA package in an effort to ensure P-CSBAs were equipped with these assets as well.

Our findings contribute to the larger body of evidence suggesting that private-sector approaches to health service delivery can work successfully to deliver services in poor communities, particularly when coupled with equally robust efforts to strengthen and collaborate with the public sector [39, 40], and lends credence to the growing agreement and understanding that reaching our development targets will require governments to work in collaborative partnership with private sector actors [41, 42].

These findings highlight a number of practical implications for future program designers and implementers. Crucial to this approach is the need to ensure that private sector actors are working, with robust monitoring supervision and coordination, toward the same objectives as those set by local, regional and national governments and that they are supported by robust and engaged community structures [43]. By engaging the public health system in the monitoring, supervision and support of this cadre, and by linking P-CSBA reporting to existing government reporting and review systems, this model ensures that the P-CSBA cadre complements and serves to strengthen the public system, effectively increasing service provision not just among this new cadre but among other skilled-cadres as well.

Policy makers should consider that the unique context in Sunamganj, a particularly remote region that has long struggled to recruit and retain public health service providers, and where out-of-pocket expenses on poor quality health care services were already quite high, was particularly well suited for this model. These conditions meant that public providers saw P-CSBA’s role as complementary to their own, not duplicative or competitive. P-CSBAs supported public provider’s efforts to reach out to remote communities and promoted utilization of health services, including publicly provided services. Out-of-pocket expenditures on health services, provided by unskilled providers, were already high prior to the project meaning that there was reduced risk of inequitable service delivery, an unintended but potential consequence of private service-delivery models.

These findings also illuminate where future investigation is needed around equity and sustainability of public-private approaches. While a preliminary analysis of our evaluation and survey data suggests that P-CSBAs were consistent sources of health services for lower income households (those in the lowest quintile were most likely to have received maternal services from a P-CSBA) more research is needed to understand the implications of a private-sector approach in such a setting [44]. Understanding not just if and how private sector providers, including P-CSBAs, are able to not just increase access to maternal health services but also how they can play a role in providing care to the poorest and most marginalized women in these communities, and thus serve as a powerful tool towards the achievement of universal health care, is an essential next step.

With these encouraging initial results, we are also intentionally exploring the sustainability of this model. Our monitoring data suggests that P-CSBAs are able to become financial sustainable with more than half earing at least 5,000 BDT (about 60 USD) per month after about one year of operations. While scaling this model from the original 50 unions to an additional 37 unions, CARE is shifting the nature of its support, focusing on the establishment of a P-CSBA self-governing structure and more robust P-CSBA network. Through this effort we hope to understand how this model can achieve not just financial sustainability but institutional sustainability allowing for more efficient coordination with public providers and the government health system with minimal external support.

### Strengths and limitations

Our ability to assess true coverage of maternal health services in the region we covered is limited because the findings presented in this article rely on self-report of services received and complications experienced which may be unreliable and subject to recall or social desirability bias.

Differences in key demographic characteristics were observed between baseline and follow-up samples. While these differences are largely reflective of documented, national and regional trends their presence makes it difficult to reliably compare across these two groups. We controlled for these changes by including a comprehensive set of demographic characteristics in our model.

Finally, because we lack a comparison group, we also face challenges in our ability to attribute the increase in service use to our efforts alone. It was not within the scope of this program to create such a comparison group during the mid-line follow-up although one is planned for the endline evaluation.

## Conclusions

Despite the fact that, in the past, the use of CSBAs in Bangladesh has not dramatically increased the proportion of births attendant by skilled attendants our findings demonstrate that, by adapting the original CSBA model to a public private partnership model; ensuring health providers were selected through a transparent process that engaged the community and local government; guaranteeing that P-CSBAs would be based in and serve their own communities; collaborating with existing public health system structures throughout for technical capacity building, logistical support, mentoring and monitoring; and providing business and entrepreneurship training in addition to clinical skills, a previously underserved community can experience dramatic increases in maternal health service utilization and reductions in self-reported complications related to pregnancy.

## Supporting information

S1 File Baseline survey tool. (PDF)

S2 File Midline survey tool. (PDF)

S3 File P-CSBA Shirpa. Shirpa was trained as a P-CSBA and now provides health services to her small village and surrounding community. Because Shirpa’s village is surrounded by water she must take a boat every morning to make her household visits.
(JPG)

S4 File Dataset. (XLSX)
