# Supplementary material for: Filling the human resource gap through public-private partnership: Can private, community-based skilled birth attendants improve maternal health service utilization and health outcomes in a remote region of Bangladesh?
Source: PLoS One. 2020 Jan 17;15(1):e0226923. doi: 10.1371/journal.pone.0226923 (PMC6968857; doi:10.1371/journal.pone.0226923)
Supplement: S1 File — (PDF) [file pone.0226923.s001.pdf]

Survey questionnaire for 'Midline study on GSK supported Community Health Worker (CHW) initiative'

Consent Form (Survey with Mother) [In English]

Protocol Title : Midline study on GSK supported Community Health Worker (CHW) initiative

Protocol No **:** PR-15124

Principal Investigator **:** Bidhan Krishna Sarker

Organization **t** International Centre for Diarrhoeal Disease Research, Bangladesh (icddr,b)

Purpose of the research: I/We have (please tell your name) come from an international research organization icddr,b which is situated in Mohakhali, Dhaka (known as Cholera Hospital). We are currently conducting a survey on maternal and child health in CARE Bangladesh project areas that will help us to know the current situation of maternal and child health in your area. Based on the result of the survey, government and related different organizations can take necessary steps in future. You may know that, in our country maternal, infant and child mortality rates are still high compared to other countries, at the same time, compared to the other districts of Bangladesh the rate of maternal and child death is the highest in Sunamganj. Correct information given by you will play vital role to know the causes of maternal and child death and that will be helpeful to reduce the maternal and child death as well as to facilitate the health of the mother and child.

Cuases of Selection: You have been selected as a participant for the study because you delivered a live birth baby in last one year and you live in this area in last 6 months where CARE Bangladesh, differnt government and non government projects are being implemented. Your active participation and in-depth knowledge share will enrich our current study and guide to future direction.

Our expectation from you: I would like to invite you to take part in an interview in our survey, which will take about one hour. You will be asked questions about maternal, neonatal, child health, family planning, nutrition, non communicable diseases and community support system related knowledge and practices. I assure you that the information you provide will only be utilized for this study.

Risk and benefits: By participating in this study, you will not be subjected to any potential risk. Though, we are unable to pay you any money for your participation for now but based on your participationa and correct information, many projects will be undertaken in this area in future, as a result you or future mother of your area will be benifited.

Privacy, anonymity and confidentiality: You can be assured that your participation and all information given by you will be kept strictly confidential and anonymous. I need your kind cooperation and free and honest discussions. Please be sure that there will be no need and no chance to identify you after this interview. The data will be kept under the confidential and strict supervision of the head of the project under lock and key. None other than the investigators of this research would have an access to the information. Any personal information related to your identity would be kept completely secret and only total scenario and results of the mother in this area will be presented. You would be able to talk to any of our project staff if you want and we are obliged to answer any questions.

Future use of information: In future, the findings of this project will be utilized for scientific knowledge generating activities and other researchers would have access to findings of this study.

Right not to participate and withdraw: Your participation in the interview is completely voluntary. It is you and only you who will decide whether you participate in this study. You can stop any time without any obligation during our discussion if you feel uncomfortable. Refusal to take part in or withdrawal from the interview will involve no penalty and no loss of your services. We can go to any private place according to your choice or if you agree, I can make arrangements so that we can sit and freely talk with privacy. You can talk in your free time, as I have no time to pressure. In case of any need, we may sit again if you permit.

We will happily provide you further information about the study, if any, now or at a later time. You may communicate with the principal investigators of the study or her/his designated person at the contact address given below. We will answer to your question related to this study. If you kindly give me your consent, then and only then I can begin the interview.

If you agree to our proposal of enrolling you in our study, please indicate that by putting your signature or your left thumb impression at the specified space below. Beside these, at the end of the interview, i will give you a copy of this consent form.

Thank you for your cooperation

_______________________________________ _____________________

Signature or left thumb impression of participant Date

_______________________________________ _____________________

Signature or left thumb impression of Parent/ Guardian/ Attendant Date

_______________________________________ _____________________

Signature or left thumb impression of the witness Date

_______________________________________ _____________________

Signature of the PI or his/her representative Date

(NOTE: In case of representative of the PI, she/he shall put her/his full name and designation and then sign)

Please contact here for further queries:

Bidhan Krishna Sarker<br>Assistant Scientis<br>MCHD, icddr,b<br>Mohakhali, Dhaka 1212,<br>Phone: 880-2-9827001-10 Ext.2266<br>E-mail: bidhan@icddrb.org	M A Salam Khan<br>IRB Secretariat<br>icddr,b<br>Mohakhali, Dhaka 1212,<br>Phone: 2- 988-6498 PABX 8860523-32 Extension. 3206<br>E-mail: salamk@icddrb.org

Starting time of interview: ☐☐ ☐☐
Hour Mins

Starting date of interview: ☐☐ ☐☐ ☐☐☐☐
Day Month Year

ID number of respondent: ☐☐☐☐ Cluster number: ☐☐☐

Screening questions

1	Did any woman in your family have live birth in last one year?	1= Yes<br>2 = No	If all the responses are yes then she will be selected for interview
2	Can she speak and listen to?	1= Yes<br>2 = No	
3	Has she been living in this area for last 6 months?	1= Yes<br>2 = No	
4	Can she give an interview now or in comming two days?	1= Yes<br>2 = No	

Section-1: Respondent's identity

No.	Question	Responses	Code	Direction
101	Name of the respondent			
102	Name of the husband			
103	Household name and number (If available)			
104	Name of village/mouza			
105	Name of ward (current)			Use code according to the planning of the study
106	Name of union and code			
107	Name of upazila and code			
**Contact number and location in detail**				
108	Contact number (cell or telephone):			
109	Contact person of the given number:			
110	Relationship of contact person with respondent			
111	Write down detail location of the household with landmark			

Section-2: Basic Household characteristics

No.	Question	Responses	Code	Direction
201	What is the main source of drinking water for members of your household?	1 = Supply water<br>2 = Tube well water<br>3 = Dug well<br>4 = Rainwater<br>5 = Pond/River/Canal<br>6 = Bottled water/Mineral Water<br>8 = Others (Specify-------------------------------)		
202	Do you boil the water to make it safer to drink?	1= Yes<br>2 = No		
203	What is the main source of water used by your household for other purposes such as cooking and handwashing?	1 = Supply water<br>2 = Tube well water<br>3 = Dug well<br>4 = Rainwater<br>5 = Pond/River/Canal<br>6 = Bottled water/Mineral Water<br>8 = Others (Specify-------------------------------)		
204	What kind of toilet facility do members of your household usually use?	1 = Sanitary latrine (linked with sewarage)<br>2 = Sanitary latrine (linked with septic tank)<br>3 = Pit latrine with slab<br>4 = Pit latrine without slab<br>5 = Open pit<br>6 = Bucket toilet<br>7 = Hanging toilet<br>8 = Others (Specify)-------------------------<br>9 = No facility/ Bush/open space/field		
205	Do you share this toilet facility with other household?	1= Yes<br>2 = No<br>3 = Not applicable		
206	What type of salt do your household mostly use for eating or cooking?	1 = Packet salt<br>2 = Open salt -------go to 208 question		
207	If used packet salt, which company salt do you use mostly?	1 = Molla Salt<br>2 = ACI salt<br>3 = Fresh salt<br>4 = Pure salt<br>5 = Pubali salt<br>6 = Teer salt<br>7 = Ifad salt<br>8 = Others (Specify-------------------------)<br>9 = Don’t know the name		
208	Does the salt have iodine that you use?	1= Yes<br>2 = No<br>9 = Don’t know		

No.	Question	Responses	Code	Direction
209	Does your household have any of the following items which are functioning (Household's own property)?			
	a) Electricity	1 = Yes 2 = No		
	b) Radio/Casset/CD player/DVD player	1 = Yes 2 = No		
	c) Television	1 = Yes 2 = No		
	d) Computer	1 = Yes 2 = No		
	e) Mobile phone	1 = Yes 2 = No		
	f) Land phone	1 = Yes 2 = No		
	g) Refrigerator	1 = Yes 2 = No		
	h) Almirah/wardrobe/ showcase/ Alna	1 = Yes 2 = No		
	i) Table	1 = Yes 2 = No		
	j) Chair	1 = Yes 2 = No		
	k) Bed	1 = Yes 2 = No		
	l) Wall clock	1 = Yes 2 = No		
	m) Bicycle	1 = Yes 2 = No		
	n) Motorcycle/motor scooter/tempo	1 = Yes 2 = No		
	o) Animal-drawn cart (cart drawn by horse)	1 = Yes 2 = No		
	p) Car/ truck	1 = Yes 2 = No		
	q) Boat	1 = Yes 2 = No		
	r) Boat with a motor	1 = Yes 2 = No		
	s) Shallow machine	1 = Yes 2 = No		
	t) Ricksha/van	1 = Yes 2 = No		
	u) Tractor	1 = Yes 2 = No		
	v) Others (Specify--------------------------)	1 = Yes 2 = No		
210	What type of fuel does your household mainly use for cooking?	01 = Electricity<br>02 = LPG/Cylinder gas<br>03 = Natural Gas/Supply Gas<br>04 = Biogas<br>05 = Kerosene<br>06 = Coal/Lignite<br>07 = Charcoal<br>08 = Wood<br>09 = Straw/Shrubs/grass<br>10 = Agricultural crop<br>11 = Animal dung<br>88 = Others (Specify-----------------)		
211	What are the construction materials of different parts of your house?			
	a. Main material of the roof	1 = Pucca (Bricks and Cement)<br>2 = Corrugated iron<br>3 = Tally<br>4 = Straw/Bamboo/Leaves<br>8 = Others (Specify-------------------)		
	b. Main material of the exterior walls	1 = Pucca (Bricks and Cement)<br>2 = Corrugated iron<br>3 = Clay/Mud<br>4 = Straw/Bamboo/Leaves<br>8 = Others (Specify-------------------)		
	c. Main material of the floor	1 = Pucca (Bricks and Cement)<br>2 = Semi – Pucca<br>3 = Clay/Kutcha<br>4 = Wood/Bamboo		
212	How many roms in this household are used for sleeping?	----------- Nos.		
213	Does your household have any own domestic animals/ poultry?	1= Yes<br>2 = No		If no, skip to 215 question
214	How many domestic animals/ poultry in your household?	a. Cows/buffalo		Write 00' if no animal
		b. Goats/sheep		
		c. Chickens/ducks/pegion		

No.	Question	Responses	Code	Direction
215	Does your household have any own homestead?<br>[Try to know that, does the household have own homestead in any other places, if yes then circle yes answer]	1= Yes<br>2 = No		
216	How much land (including ponds) does your household own (other than the homestead land)?	--------------------Decemal		Write 00' if no land

Section-3: Basic individual characteristics and socio-demographic information

No.	Question	Responses	Code	Direction
301	What is your age?	------ --------<br>Year Month		
302	What is your religion?	1 = Islam<br>2 = Hindu<br>3 = Christian<br>4 = Buddhist<br>8 = Other (Specify..)		
303	What is your completed year(s) of education?	--------------- Completed year(s) of education		Write 00 if no institutional education
304	What is your husband's completed year of education?	--------------- Completed year(s) of education		
305	What is your current marital status?	1 = Currently married<br>2 = Divorced<br>3 = Separated<br>4 = Widowed<br>5 = Deserted<br>6 = Never married		
306	What is your primary occupation?	01 = Service (Govt.)<br>02 = Service (Non govt.)<br>03 = Business<br>04 = Agriculture/Farming/Fisherman<br>05 = Handicraft<br>06 = Rickshaw/Van puller/Boat man<br>07 = Motorized transport worker<br>08 = Skilled worker (Goldsmith/Masion/ Carpenter/Electrician)<br>09 = Student<br>10 = Housewife<br>11 = Unemployed<br>12 = Expatriate<br>13 = Day labour<br>88 = Others (specify--------------------)<br>77 = Not applicable		
307	Are you currently involved in any kind of income generating activities?	1= Yes<br>2 = No		

No.	Question	Responses	Code	Direction
308	What is your husband's occupation?	01 = Service (Govt.)<br>02 = Service (Non govt.)<br>03 = Business<br>04 = Agriculture/Farming/Fisherman<br>05 = Handicraft<br>06 = Rickshaw/Van puller/Boat man<br>07 = Motorized transport worker<br>08 = Skilled worker (Goldsmith/Masion/ Carpenter/Electrician)<br>09 = Student<br>10 = Unemployed<br>11 = Elderly (60 years or more)/Retired/ Disable<br>12 = Expatriate<br>13 = Day labour<br>88 = Others (specify--------------------)<br>77 = Not applicable		
309	How many members do you have in your family?	--------------------------------------- Nos.		
310	How many of your family members involved in income generation?	-- Nos.		
311	What is the main source of income in your family?	01 = Service (Govt.)<br>02 = Service (Non govt.)<br>03 = Business<br>04 = Agriculture/Farming/Fisherman<br>05 = Handicraft<br>06 = Rickshaw/Van puller/Boat man<br>07 = Motorized transport worker<br>08 = Skilled worker (Goldsmith/Masion/ Carpenter/Electrician)<br>09 = Expatriate<br>10 = Day labour<br>88 = Others (specify--------------------)		
312	What is your individual average monthly income?	--------------------------------------- Tk.		Write '00' if no income
**Reproductive and obstetric information**				
313	At what age were you married for the first time?	---------------------- Age in completed years		
314	How many times were you become pregnant?	---------------------- times		
314.a	How many children have you ever given birth?	---------------------- times		
315. a	How many living children do you have now?	---------------------- Nos.		
315. b	How many under 5 years living children do you have now?	---------------------- Nos.		

Pregnancy History:

316	a) Pregnancy	b) Outcome of pregnancies	c) If live birth then date of birth		d) Gender	e)Children currently dead or alive	f) If alive, currently how old is he/she? If can't remember write 9999	Only ask for 2-5 years child<br>**g)** Did the baby breastfeed minimum 2 years?	Only ask for under 2 years child<br>h) Did the baby undergo GMP to understand whether the growth of the baby is in the right direction	i) If still birth, Date of death	j) Cause of death (Use below code)
316a	1st		A	----/-----/-----<br>Day/Month/Year			---- -----<br>Year Month	1=Yes, 2= No	1=Yes, 2= No	----/-----/-----<br>Day/Month/Year	
			B	----/-----/-----<br>Day/Month/Year			---- -----<br>Year Month	1=Yes, 2= No	1=Yes, 2= No	----/-----/-----<br>Day/Month/Year	
316b	2nd		A	----/-----/-----<br>Day/Month/Year			---- -----<br>Year Month	1=Yes, 2= No	1=Yes, 2= No	----/-----/-----<br>Day/Month/Year	
			B	----/-----/-----<br>Day/Month/Year			---- -----<br>Year Month	1=Yes, 2= No	1=Yes, 2= No	----/-----/-----<br>Day/Month/Year	
316c	3rd		A	----/-----/-----<br>Day/Month/Year			---- -----<br>Year Month	1=Yes, 2= No	1=Yes, 2= No	----/-----/-----<br>Day/Month/Year	
			B	----/-----/-----<br>Day/Month/Year			---- -----<br>Year Month	1=Yes, 2= No	1=Yes, 2= No	----/-----/-----<br>Day/Month/Year	
316d	4th		A	----/-----/-----<br>Day/Month/Year			---- -----<br>Year Month	1=Yes, 2= No	1=Yes, 2= No	----/-----/-----<br>Day/Month/Year	
			B	----/-----/-----<br>Day/Month/Year			---- -----<br>Year Month	1=Yes, 2= No	1=Yes, 2= No	----/-----/-----<br>Day/Month/Year	
316e	5th		A	----/-----/-----<br>Day/Month/Year			---- -----<br>Year Month	1=Yes, 2= No	1=Yes, 2= No	----/-----/-----<br>Day/Month/Year	
			B	----/-----/-----<br>Day/Month/Year			---- -----<br>Year Month	1=Yes, 2= No	1=Yes, 2= No	----/-----/-----<br>Day/Month/Year	
316f	6th		A	----/-----/-----<br>Day/Month/Year			---- -----<br>Year Month	1=Yes, 2= No	1=Yes, 2= No	----/-----/-----<br>Day/Month/Year	
			B	----/-----/-----<br>Day/Month/Year			---- -----<br>Year Month	1=Yes, 2= No	1=Yes, 2= No	----/-----/-----<br>Day/Month/Year	
316g	7th		A	----/-----/-----<br>Day/Month/Year			---- -----<br>Year Month	1=Yes, 2= No	1=Yes, 2= No	----/-----/-----<br>Day/Month/Year	
			B	----/-----/-----<br>Day/Month/Year			---- -----<br>Year Month	1=Yes, 2= No	1=Yes, 2= No	----/-----/-----<br>Day/Month/Year	
316h	8th		A	----/-----/-----<br>Day/Month/Year			---- -----<br>Year Month	1=Yes, 2= No	1=Yes, 2= No	----/-----/----<br>Day/Month/Year	
			B	----/-----/-----<br>Day/Month/Year			---- -----<br>Year Month	1=Yes, 2= No	1=Yes, 2= No	----/-----/-----<br>Day/Month/Year	
316i	9th		A	----/-----/-----<br>Day/Month/Year			---- -----<br>Year Month	1=Yes, 2= No	1=Yes, 2= No	----/-----/-----<br>Day/Month/Year	
			B	----/-----/-----<br>Day/Month/Year			---- -----<br>Year Month	1=Yes, 2= No	1=Yes, 2= No	----/-----/-----<br>Day/Month/Year	

b) Pregnancy outcome: 1= Live birth, 2= Still birth , 3= MR/D&C/Miscarriage/Abortion, 4 = Currently pregnant
d) Gender: 1= Son, 2= Daughter
e) Current status of the child: 1 = Alive, 2 = Death
j) Causes of death: 01 = Pneumonia, 02 = Jaundice, 03 = Drowning, 04 = Diarrhoea, 05 = Delivery related infection, 06 = Navel infection, 07 = Ghost/Jin/Bad air, 08 = High fever, 09 = Convulsion , 10 = Malnutrition, 88 = Others (Details----------------------------), 99 = Don't know

No.	Question	Responses	Code	Direction
317	What was the date of your last delivery?	------ -------- ---------<br>Day Month Year<br>9999= Can't say/Don't know		
318	What was the mode of your last delivery	1= Normal delivery<br>2= Caesarean delivery<br>8= Others (sprcify----------------)		
319	Are you pregnant now?	1 = Yes<br>2 = No		
320	Do you wish to take another child in next two years?	1 = Yes<br>2 = No		
321	At the last time you became pregnant, did you want to become pregnant at that time or wanted to wait and have pregnancy later or never wanted to have any more children?	1 = Wanted at that time<br>2 = Wanted later<br>3 = Never wanted any more children		

Section-4: MNCH FP, Non Communicable Diseases and Nutrition related Knowledge question

No.	Question		Responses	Code	Direction
In this part, I would like to ask you some questions regarding pregnancy					
401	How many times a pregnant mother should go for antenatal check-ups?		----------------Times<br>99= Don't know		
402	When should a pregnant woman have her 1st ANC visits?		----------------Month<br>99= Don't know		
403	How much food a woman should take during pregnancy?		1 = Normal<br>2 = More than normal<br>3 = Less than normal<br>9= Don't know		
404	Can you say that what are the danger signs of pregnancy?<br><br>[Probe for multiple answer]	a) Complication during pregnancy<br><br>[Probe for multiple answer]	01 = Excessive bleeding/ Hemorrhage<br>02= Convulsion<br>03= Severe Headache<br>04= High Fever<br>05= Blurring of vision<br>06= Swollen legs/hands/face(edema)<br>07= High blood pressure<br>08= Reduced/absent fetal movement<br>09= Foul smelling discharge from vagina<br>10= Sever anemia<br>11= Rupture of membrane before 37 weeks(PROM)<br>88= Others<br>99= Do not know		
		b) Delivery complication<br><br>[Probe for multiple answer]	01= Excessive bleeding/Hemorrhage<br>02= Labor lasting more than 12 hours<br>03= Hand/leg prolapsed/ breach presentation<br>04= Foul smelling discharge from vagina<br>05= High fever<br>06= Retained placenta<br>07= Convulsion<br>08= Tetanus<br>09= Severe lower abdominal pain<br>10= Severe chest pain and rapid breathing<br>88= Others<br>99= Do not know		

		c) Post delivery complication<br><br>[Probe for multiple answer]	01 = Excessive bleeding /Hemorrhage<br>02 = Convulsion/ fits<br>03 = High fever<br>04 = Foul smelling discharge from vagina<br>05 = Wound infections<br>06 = Inverted nipples<br>07 = Engorged breast<br>08 = Tetanus<br>09 = Severe lower abdominal pain<br>10 = Severe chest pain and rapid breathing<br>11 = Uterine prolapse<br>88 = Other (Specify)---------------<br>99 = Do not know		
405	By whom or how did you know about these complication (During pregnancy, Delivery, Post delivery)?<br><br>[Probe for multiple answer]		01= Trditional Birth Attendant<br>02= Relatives/Neighbors<br>03= Trained Traditional Birth Attendant (Govt.)<br>04= Trained Traditional Birth Attendant (Non govt.)<br>05= SACMO/MA (Govt.)<br>06= Nurse/ Midwife/FWV (Govt.)<br>07= Nurse/ Midwife/FWV (Non-govt.)<br>08= Qualified doctor (MBBS)<br>09= VD/Pharmacist<br>10= NGO health worker (Detail.....................)<br>11= PCSBA<br>12= CHW<br>13= CmSS members<br>14= Up members<br>15= CHCP<br>16= Media (TV/Radio/Daily newspapers)<br>17= Poster/Banner/Signboard/Brochure/Leaflet<br>18= FWA/HA<br>88= Others (Detail.....................)<br>99= Do not know		
In this part, I would like to ask you some qeustions regarding delivery					
406	According to you, where should a woman have her delivery?		01 = At home<br>02= Rural Dispensary (RD)<br>03= Family Welfare Center (FWC)<br>04= Union Health & Family Welfare Center (UH&FWC)<br>05= Upazila Health Complex (UHC)<br>06= Maternal and Child Welfare Center (MCWC)<br>07= District Hospital (DH)<br>08= Medical College Hospital (MCH)<br>09= Community Clinic (CC)<br>10 = Private Hospital/Clinic (Profitable)<br>11 = Private Hospital (Non profitable/NGO)<br>88= Others (Detail.....................)<br>99= Do not know		
407	In your opinion, who should conduct the delivery?		01= Trditional Birth Attendant<br>02= Relatives/Neighbors<br>03= Trained Traditional Birth Attendant (Govt.)<br>04= Trained Traditional Birth Attendant (Non govt.)<br>05= SACMO/Medical Assistant (MA) (Govt.)<br>06= Nurse/ Midwife/FWV (Govt.)<br>07= Nurse/ Midwife/FWV (Non-govt.)<br>08= Qualified doctor (MBBS)<br>09= VD/Pharmacist<br>10= NGO health worker (Detail.....................)<br>11= PCSBA<br>12= CHCP<br>88= Others (Detail.....................)<br>99= Do not know		

In this part, I would like to ask you some questions regarding post delivery				
408	How many times a pregnant mother should go for postnatal check-ups?	----------------Times<br>99= Don’t know		
409	When should a pregnant woman have her 1st PNC?	----------Day------------Hour<br>99= Don’t know		
In this part, I would like to ask you some questions regarding family planning methods				
410	Which methods have you heard about?<br><br>[Probe for multiple answer]	01= Female sterilization<br>02 = Male sterilization<br>03 = Pill<br>04 = Intrauterine devices (IUD)<br>05 = Injectables<br>06 = Implant/Norplant<br>07 = Condom<br>08 = Safe period<br>09 = Withdrawl<br>88 = Other (specify ----------------------------------)		
411	By whom or how did you know about these methods?<br><br>[Probe for multiple answer]	01= Trditional Birth Attendant<br>02= Relatives/Neighbors<br>03= Trained Traditional Birth Attendant (Govt.)<br>04= Trained Traditional Birth Attendant (Non govt.)<br>05= SACMO/MA (Govt.)<br>06= Nurse/ Midwife/FWV (Govt.)<br>07= Nurse/ Midwife/FWV (Non-govt.)<br>08= Qualified doctor (MBBS)<br>09= VD/Pharmacist<br>10= NGO health worker (Detail.....................)<br>11= PCSBA<br>12= CHW<br>13= CmSS members<br>14= Up members<br>15= CHCP<br>16= Media (TV/Radio/Daily newspapers)<br>17= Poster/Banner/Signboard/Brochure/Leaflet<br>18= FWA/HA<br>88= Others (Detail.....................)<br>99= Do not know		
In this part, I would like to ask you some questions regarding adolescent health				
412	In your opinion, what kind of information or services related to adolescent health is needed for adolescent girls in your areas?<br><br>[Probe for multiple answer]	01= Provide advice and service on reproductive health<br>02= Necessary advise and treatment given in prevention of STD<br>03= Provide TT immunization<br>04= Provide IFA tablet<br>05= Provide information and education equipment related to clenliness during menstruation<br>06= Provide advice and service related to nutrition<br>07= Awareness build up related to child marriage<br>08= Provide advice related to adolescent pregnancy<br>09= Create awareness and provide advise to prevent the drug use<br>10= Arrangement of available sanitary napkin<br>88= Others (Detail.....................)		
413	Can you say that, what kind of problems might happen if married adolescent girl under 19 years age become pregnant?<br><br>[Probe for multiple answer]	1 = No problem<br>2 = Health risk of mother<br>3 = Health risk of baby<br>4 = Education might get stopped<br>5 = Might not continue job<br>8= Others (Detail.....................)<br>9= Do not know		

In this part, I would like to ask you some questions regarding newborn and child health				
414	Could you name a few basic essential newborn cares that can be provided to baby just after delivery?<br>[Probe for multiple answer]	01 = Drying and wrapping with clean and warm cloth<br>02 = Start breast Feeding /colostrum within 1 hour of delivery<br>03 = Cut the cord with clean blade<br>04 = Taking care of eyes<br>05 = Keep the cord of the baby dry/ cord care<br>06 = Give the newborn a bath at least after 72 hours<br>07 = Avoid shaving during the first month<br>08 = Take special care of low birth weight/ pre term baby<br>09 = Refer if any complication observed<br>88 = Other (specify) --------------------------------<br>99 = Do not know		
415	What are the danger signs for a newborn baby?<br>[Probe for multiple answer]	01 = Weak cry<br>02 = Absent cry<br>03 = Unable to suck or breast feed<br>04 = Lethargy<br>05 = Cold hands and feet<br>06 = Rapid breathing<br>07 = Chest indrawing<br>08 = High temparature (fever)<br>09 = Convulsion/unconsciousness<br>10 = Umbilical discharge<br>11 = More than 10 skin pustules<br>12 = Red eyes with discharge<br>13 = Jaundice for more than 14 days<br>14 = Low temparature<br>88 = Other ------------------------------<br>99 = Do not know		If answers is don't know then go to 501 question
416	By whom or how did you know about these danger signs?<br><br>[Probe for multiple answer]	01= Trditional Birth Attendant<br>02= Relatives/Neighbors<br>03= Trained Traditional Birth Attendant (Govt.)<br>04= Trained Traditional Birth Attendant (Non govt.)<br>05= SACMO/MA (Govt.)<br>06= Nurse/ Midwife/FWV (Govt.)<br>07= Nurse/ Midwife/FWV (Non-govt.)<br>08= Qualified doctor (MBBS)<br>09= VD/Pharmacist<br>10= NGO health worker (Detail.....................)<br>11= PCSBA<br>12= CHW<br>13= CmSS members<br>14= Up members<br>15= CHCP<br>16= Media (TV/Radio/Daily newspapers)<br>17= Poster/Banner/Signboard/Brochure/Leaflet<br>18= FWA/HA<br>88= Others (Detail.....................)<br>99= Do not know		

Section-5: Pregnancy Registration, Birth planning, ANC, DC and PNC related practice questions

No.	Question	Responses	Code	Direction
**Pregnancy Registration and Birth Planning**				
501	Were you registered or listed at the time of your pregnancy by any healthcare provider or organization?	1 = Yes<br>2 = No		If no then go 503
502	If you were registered then, by whom were you registered?<br><br>[Probe for multiple answer]	01= Trditional Birth Attendant<br>02= Relatives/Neighbors<br>03= Trained Traditional Birth Attendant (Govt.)<br>04= Trained Traditional Birth Attendant (Non govt.)<br>05= SACMO/MA (Govt.)<br>06= Nurse/ Midwife/FWV (Govt.)<br>07= Nurse/ Midwife/FWV (Non-govt.)<br>08= Qualified doctor (MBBS)<br>09= VD/Pharmacist<br>10= NGO health worker (Detail.....................)<br>11= PCSBA<br>12= CHW<br>13= CmSS members<br>14= Up members<br>15= CHCP<br>16= FWA/HA<br>88= Others (Detail.....................)<br>99= Do not know		
503	Did any one discuss with you/your family about birth planning/birth preparedness in your last pregnancy? (Explain birth preparedness)	1 = Yes<br>2 = No		If no then go 508
504	If yes then, who discussed about birth preparedness with you?<br><br>[Probe for multiple answer]	01= Trditional Birth Attendant<br>02= Relatives/Neighbors<br>03= Trained Traditional Birth Attendant (Govt.)<br>04= Trained Traditional Birth Attendant (Non govt.)<br>05= SACMO/MA (Govt.)<br>06= Nurse/ Midwife/FWV (Govt.)<br>07= Nurse/ Midwife/FWV (Non-govt.)<br>08= Qualified doctor (MBBS)<br>09= VD/Pharmacist<br>10= NGO health worker (Detail.....................)<br>11= PCSBA<br>12= CHW<br>13= CmSS members<br>14= Up members<br>15= CHCP<br>16= FWA/HA<br>88= Others (Detail.....................)<br>99= Do not know		
505	What were the topics discussed on the session about birth preparedness?<br><br>[Probe for multiple answer]	01= The delivery place selection<br>02= Savings money<br>03= Arranging transport in emergency<br>04= Birth attendant selection<br>05= Blood donor selection<br>06= About the referral facilities if any complication arise<br>07= About the accompanying person if complications arise<br>08= Save delivery kit collection<br>88= Others<br>99= Don’t know		

No.	Question	Responses	Code	Direction
506	Which family members/you participated on the discussion?<br><br>[Probe for multiple answer]	01= Own<br>02= Husband<br>03= Father-in-law<br>04= Mother-in-law<br>05= Mother<br>06= Father<br>07= Brother-in-law<br>08= Sister-in-law<br>09= Brother/Sister<br>10= Aunt<br>88= Others (Detail.....................)		
507	During your last pregnancy, how many times the discussion related to birth planning happened?	----------times		
508	During your last pregnancy, did anyone provide birth planning card to you or your family members?	1 = Yes<br>2 = No		If possible show the card to know the answer,<br>If no then go 510
509	If yes, then who provided the birth planning card?	01= Trditional Birth Attendant<br>02= Relatives/Neighbors<br>03= Trained Traditional Birth Attendant (Govt.)<br>04= Trained Traditional Birth Attendant (Non govt.)<br>05= SACMO/MA (Govt.)<br>06= Nurse/ Midwife/FWV (Govt.)<br>07= Nurse/ Midwife/FWV (Non-govt.)<br>08= Qualified doctor (MBBS)<br>09= VD/Pharmacist<br>10= NGO health worker (Detail.....................)<br>11= PCSBA<br>12= CHW<br>13= CmSS members<br>14= Up members<br>15= CHCP<br>16= FWA/HA<br>88= Others (Detail.....................)<br>99= Do not know		

No.	Question	Responses	Code	Direction
**Antenatal care:**				
510	Did you receive any antenatal check up during your last pregnancy?	1 = Yes<br>2 = No		IF yes, then ask the following question in the table other wise skip to q.513
511	If yes, How many times did you have check-ups/ antenatal care during your last pregnancy?	----------------------times<br>99 =Can't say/Don't know		

512	a) Antenatal check-ups	b) Place	c) Provider	d) Gestational age of receiving care (Week)	e) Components tested during ANC (Ask each of the code and write down)	f) Cost of services	Direction
512a	ANC-1			☐☐			Use the given code for place, provder and test code
512b	ANC-2			☐☐			
512c	ANC-3			☐☐			
512d	ANC-4			☐☐			
512e	Last ANC			☐☐			

b) Place	c) Providers	e) Test code
01 = At home<br>02= Community Clinic (CC)<br>03= Rural Dispensary (RD)<br>04= Family Welfare Center (FWC)<br>05= Union Health & Family Welfare Center (UH&FWC)<br>06= Upazila Health Complex (UHC)<br>07= Maternal and Child Welfare Center (MCWC)<br>08= District Hospital (DH)<br>09= Medical College Hospital (MCH)<br>10 = Private Hospital (Non profitable/NGO)<br>11 = Private Hospital/Clinic (Profitable)<br>12 = Doctor's private chamber<br>13 = Pharmacy or Drug shop<br>88= Others (Detail.....................)	01= Trditional Birth Attendant<br>02= Relatives/Neighbors<br>03= Trained Traditional Birth Attendant (Govt.)<br>04= Trained Traditional Birth Attendant (Non govt.)<br>05= SACMO/MA (Govt.)<br>06= Nurse/ Midwife/FWV (Govt.)<br>07= Nurse/ Midwife/FWV (Non-govt.)<br>08= Qualified doctor (MBBS)<br>09= VD/Pharmacist<br>10= NGO health worker (Detail.....................)<br>11= PCSBA<br>12= CHCP<br>13= FWA/HA<br>14= Homeo doctor<br>88= Others (Detail.....................)<br>99= Do not know/Can't remember	01 = Height<br>02 = Weight<br>03 = Check anemia<br>04 = Check Edima<br>05 = Check blood pressure<br>06 = Check belly<br>07 = Ultrasonogram<br>08 = Blood test<br>09 = Urine test<br>10 = Provide advice<br>88= Others (Detail................)

No.	Question	Responses	Code	Direction
513	What kind of complications did you experience in your last pregnancy?<br><br>[Probe for multiple answer]	01 = Excessive bleeding/ Hemorrhage<br>02= Convulsion<br>03= Severe Headache<br>04= High Fever<br>05= Blurring of vision<br>06= Swollen legs/hands/face(edema)<br>07= High blood pressure<br>08= Reduced/absent fetal movement<br>09= Foul smelling discharge from vagina<br>10= Sever anemia<br>11= Rupture of membrane before 37 weeks(PROM)<br>88= Others (Details..............................)<br>55= No complication	☐☐<br>☐☐<br>☐☐<br>☐☐<br>☐☐<br>☐☐<br>☐☐	If no complication then go to 522

No.	Question	Responses	Code	Direction
514	If yes, to which provider did you/your family member went/contacted first for that complication?	01= Trditional Birth Attendant<br>02= Relatives/Neighbors<br>03= Trained Traditional Birth Attendant (Govt.)<br>04= Trained Traditional Birth Attendant (Non govt.)<br>05= SACMO/MA (Govt.)<br>06= Nurse/ Midwife/FWV (Govt.)<br>07= Nurse/ Midwife/FWV (Non-govt.)<br>08= Qualified doctor (MBBS)<br>09= VD/Pharmacist<br>10= NGO health worker (Detail.....................)<br>11= PCSBA<br>12= CHW<br>13= CmSS members<br>14= Up members<br>15= CHCP<br>16= FWA/HA<br>17= Homeo doctor<br>18= Didn't contact with anybody<br>88= Others (Detail.....................)<br>99= Do not know		If ans is didn't contact with anybody then go to 522
515	Did the provider refer you for receiving treatment?	1 = Yes<br>2 = No		If don't refer then go 521
516	Where did the provider refer you?	01 = At home<br>02= Community Clinic (CC)<br>03= Rural Dispensary (RD)<br>04= Family Welfare Center (FWC)<br>05= Union Health & Family Welfare Center (UH&FWC)<br>06= Upazila Health Complex (UHC)<br>07= Maternal and Child Welfare Center (MCWC)<br>08= District Hospital (DH)<br>09= Medical College Hospital (MCH)<br>10 = Private Hospital (Non profitable/NGO)<br>11 = Private Hospital/Clinic (Profitable)<br>12 = Doctor's private chamber<br>13 = To PCSBA<br>88= Others (Detail.....................)<br>99= Don't know		
517	Did you go the refered place for taking treatment?	1 = Yes<br>2 = No		If don't go refered place then go 521
518	At last, from where did you take the treatment?	01 = At home<br>02= Community Clinic (CC)<br>03= Rural Dispensary (RD)<br>04= Family Welfare Center (FWC)<br>05= Union Health & Family Welfare Center (UH&FWC)<br>06= Upazila Health Complex (UHC)<br>07= Maternal and Child Welfare Center (MCWC)<br>08= District Hospital (DH)<br>09= Medical College Hospital (MCH)<br>10 = Private Hospital (Non profitable/NGO)<br>11 = Private Hospital/Clinic (Profitable)<br>12 = Doctor's private chamber<br>13 = To PCSBA<br>88= Others (Detail.....................)<br>99= Don't know		

519	If you are referred, during refer, who helped you in different stages?<br><br>[Probe for multiple answer]	01= Trditional Birth Attendant<br>02= Relatives/Neighbors<br>03= Trained Traditional Birth Attendant (Govt.)<br>04= Trained Traditional Birth Attendant (Non govt.)<br>05= SACMO/MA (Govt.)<br>06= Nurse/ Midwife/FWV (Govt.)<br>07= Nurse/ Midwife/FWV (Non-govt.)<br>08= Qualified doctor (MBBS)<br>09= VD/Pharmacist<br>10= NGO health worker (Detail.....................)<br>11= PCSBA<br>12= CHW<br>13= CmSS members<br>14= Up members<br>15= CHCP<br>16= FWA/HA<br>17= Homeo doctor<br>88= Others (Detail.....................)<br>99= Do not know		
520	During refer, how did they/he/she help you?<br><br>[Probe for multiple answer]	01= Selecting the facility and service provider<br>02= Arranging transport to go to health facility<br>03= Taking me to the health facility<br>04= Helping me to take treatment from the facility<br>05= Communicating with referral facility<br>06= Giving money<br>88= Others (Details---------------------------)<br>99= Don't know		
521	During pregnancy period, total how much money did you have to spend for health complications?	---------------Tk.		
**Delivery Care**				
522	What preparation did you take for your last delivery?<br><br>[Probe for multiple answer]	01= Decided about place of delivery<br>02= Saved money<br>03= Prepared emergency transport<br>04= Decided about birth attendant<br>05= Selected about blood donor<br>06= Selected referral facilities if any complication<br>07= Accompanying person for complications<br>08= Collected save delivery kit<br>88= Others (Details.................................)<br>99= Did not take any preparation		If delivery place decided beforehand then ask next question otherwise go to 524
523	If delivery place decided beforehand then where did you want to deliver your baby?	01 = Own home (With know TBA)<br>02 = Parent's home<br>03 = In-laws home<br>04= Community Clinic (CC)<br>05= Rural Dispensary (RD)<br>06= Family Welfare Center (FWC)<br>07= Union Health & Family Welfare Center (UH&FWC)<br>08= Upazila Health Complex (UHC)<br>09= Maternal and Child Welfare Center (MCWC)<br>10= District Hospital (DH)<br>11= Medical College Hospital (MCH)<br>12 = Private Hospital/Clinic (Profitable)<br>13 = Private Hospital (Non profitable/NGO)<br>14= Own home (With PCSBA)<br>88 = Others (Details.................................)		

No.	Question	Responses	Code	Direction
524	Where was your last delivery conducted?	01 = Own home (With know TBA)<br>02 = Parent's home<br>03 = In-laws home<br>04= Community Clinic (CC)<br>05= Rural Dispensary (RD)<br>06= Family Welfare Center (FWC)<br>07= Union Health & Family Welfare Center (UH&FWC)<br>08= Upazila Health Complex (UHC)<br>09= Maternal and Child Welfare Center (MCWC)<br>10= District Hospital (DH)<br>11= Medical College Hospital (MCH)<br>12 = Private Hospital/Clinic (Profitable)<br>13 = Private Hospital (Non profitable/NGO)<br>14= Own home (With PCSBA)<br>88 = Others (Details.................................)		
525	Who conducted the delivery?	01= Trditional Birth Attendant<br>02= Relatives/Neighbors<br>03= Trained Traditional Birth Attendant (Govt.)<br>04= Trained Traditional Birth Attendant (Non govt.)<br>05= SACMO/MA (Govt.)<br>06= Nurse/ Midwife/FWV (Govt.)<br>07= Nurse/ Midwife/FWV (Non-govt.)<br>08= Qualified doctor (MBBS)<br>09= VD/Pharmacist<br>10= NGO health worker (Detail.....................)<br>11= PCSBA<br>12= CHCP<br>13= Self<br>14= FWA/HA Up members<br>88= Others (Detail.....................)<br>99= Do not know		
526	For home delivery, did you use delivery kit during your delivery?	1 = Yes<br>2 = No<br>9 = Don't know		Only ask for home delivery
527	Around the time of the birth of (Name), what kind of complications did you experience?<br><br>[Probe for multiple answer]	01= Excessive bleeding/Hemorrhage<br>02= Labor lasting more than 12 hours<br>03= Hand/leg prolapsed/ breach presentation<br>04= Foul smelling discharge from vagina<br>05= High fever<br>06= Retained placenta<br>07= Convulsion<br>08= Tetanus<br>09= Severe chest pain and rapid breathing<br>88= Others<br>55= No complication		If no complication then go to 536

528	To which provider did you/your family member went/contacted first for those complications?	01= Trditional Birth Attendant<br>02= Relatives/Neighbors<br>03= Trained Traditional Birth Attendant (Govt.)<br>04= Trained Traditional Birth Attendant (Non govt.)<br>05= SACMO/MA (Govt.)<br>06= Nurse/ Midwife/FWV (Govt.)<br>07= Nurse/ Midwife/FWV (Non-govt.)<br>08= Qualified doctor (MBBS)<br>09= VD/Pharmacist<br>10= NGO health worker (Detail.....................)<br>11= PCSBA<br>12= CHW<br>13= CmSS members<br>14= Up members<br>15= CHCP<br>16= FWA/HA<br>17= Homeo doctor<br>18= Didn't contact with anybody<br>88= Others (Detail.....................)<br>99= Do not know		If ans is didn't contact with anybody then go to 536
529	Did the provider refer you?	1 = Yes<br>2 = No		If don't refer then go 535
530	Where did the provider refer you?	01 = At home<br>02= Community Clinic (CC)<br>03= Rural Dispensary (RD)<br>04= Family Welfare Center (FWC)<br>05= Union Health & Family Welfare Center (UH&FWC)<br>06= Upazila Health Complex (UHC)<br>07= Maternal and Child Welfare Center (MCWC)<br>08= District Hospital (DH)<br>09= Medical College Hospital (MCH)<br>10 = Private Hospital (Non profitable/NGO)<br>11 = Private Hospital/Clinic (Profitable)<br>12 = Doctor's private chamber<br>13 = To PCSBA<br>88= Others (Detail.....................)<br>99= Don't know		
531	Did you go the refered place for taking treatment?	1 = Yes<br>2 = No		If don't go refered place then go 535
532	At last, from where did you take the treatment?	01 = At home<br>02= Community Clinic (CC)<br>03= Rural Dispensary (RD)<br>04= Family Welfare Center (FWC)<br>05= Union Health & Family Welfare Center (UH&FWC)<br>06= Upazila Health Complex (UHC)<br>07= Maternal and Child Welfare Center (MCWC)<br>08= District Hospital (DH)<br>09= Medical College Hospital (MCH)<br>10 = Private Hospital (Non profitable/NGO)<br>11 = Private Hospital/Clinic (Profitable)<br>12 = Doctor's private chamber<br>13 = To PCSBA<br>88= Others (Detail.....................)<br>99= Don't know		

No.	Question	Responses	Code	Direction
533	If you are referred, during refer, who helped you in different stages?<br><br>[Probe for multiple answer]	01= Trditional Birth Attendant<br>02= Relatives/Neighbors<br>03= Trained Traditional Birth Attendant (Govt.)<br>04= Trained Traditional Birth Attendant (Non govt.)<br>05= SACMO/MA (Govt.)<br>06= Nurse/ Midwife/FWV (Govt.)<br>07= Nurse/ Midwife/FWV (Non-govt.)<br>08= Qualified doctor (MBBS)<br>09= VD/Pharmacist<br>10= NGO health worker (Detail.....................)<br>11= PCSBA<br>12= CHW<br>13= CmSS members<br>14= Up members<br>15= CHCP<br>16= FWA/HA<br>17= Homeo doctor<br>88= Others (Detail.....................)<br>99= Do not know		
534	During refer, how did they/he/she help you?<br><br>[Probe for multiple answer]	01= To select the facility and service provider<br>02= Arranged transport to go to health facility<br>03= Took me to the health facility<br>04= Helped to take treatment from the facility<br>05= To communicate with referral facility<br>06= To give money<br>88= Others (Details---------------------------)<br>99= Don't know		
535	How much money did you have to spend for delivery health complication?	---------------Tk.		
536	How did your husband and other male members cooperate you in receiving health care services (ANC, Delivery, PNC) during your last pregnacy?<br><br>[Probe for multiple answer]	01= Helped to decide the health care center and health care provider<br>02= Accompanied during going to health care center<br>03= Arranged vehicles for going to the health care center<br>04= Paid the service charge<br>05= Brought the required medicines<br>88= Others(Details ----------------------------------)<br>99= Didn't cooperate		
537	In which household chores, did your husband and other male members of yours household help in your last pregnancy?<br><br>[Probe for multiple answer]	01= To cook<br>02= To domestic work<br>03= To bring water/ boil water<br>04= To look after others children<br>05= To wash/dry cloth<br>06= For shopping<br>88= Others(Details ----------------------------------)<br>99= Didn't cooperate		
538	During delivery, did you get any kind of support from other than your household members (e.g. Transport, Money, Accompany or others)?	1 = Yes<br>2 = No		If no then go to 541
539	From whom did you get the support?<br><br>[Probe for multiple answer]	1= Relative<br>2= Neighbour/Friend<br>3= CmSS members<br>4= Up members<br>5= CHW<br>8= Others(Details ----------------------------------)		

No.	Question	Responses	Code	Direction
540	What kind of support you received that time?<br><br>[Probe for multiple answer]	1 = Money<br>2 = Transportation<br>3 = Accompany<br>4 = Demand Side Financing (DSF)<br>5= Refer<br>8 = Others (specify----------------)		
541	Total how much money did you spend in your last delivery?	......................................Tk.		
**Postnatal Care**				
542	Did you receive any postnatal check up after your last delivery?	1 = Yes<br>2 = No		If no then go to 545
543	If yes, How many times did you have check-ups/ postnatal care after your last delivery?	----------------------times<br>99 =Can't say/Don't know		

544	a) Postnatal check-ups	b) Place	c) Provider	d) Service receiving time (Hour & Minutes)	e) Components test during PNC (Ask each of the code and write down)	f) Cost of services	Direction
544a	PNC-1						Use the given code for place, provder and test code
544b	PNC-2						
544c	PNC-3						
544d	PNC-4						
544e	Last PNC						

b) Place	c) Providers	e) Test code
01 = At home<br>02= Community Clinic (CC)<br>03= Rural Dispensary (RD)<br>04= Family Welfare Center (FWC)<br>05= Union Health & Family Welfare Center (UH&FWC)<br>06= Upazila Health Complex (UHC)<br>07= Maternal and Child Welfare Center (MCWC)<br>08= District Hospital (DH)<br>09= Medical College Hospital (MCH)<br>10 = Private Hospital (Non profitable/NGO)<br>11 = Private Hospital/Clinic (Profitable)<br>12 = Doctor's private chamber<br>13 = Pharmacy or Drug shop<br>88= Others (Detail.....................)	01= Trditional Birth Attendant<br>02= Relatives/Neighbors<br>03= Trained Traditional Birth Attendant (Govt.)<br>04= Trained Traditional Birth Attendant (Non govt.)<br>05= SACMO/MA (Govt.)<br>06= Nurse/ Midwife/FWV (Govt.)<br>07= Nurse/ Midwife/FWV (Non-govt.)<br>08= Qualified doctor (MBBS)<br>09= VD/Pharmacist<br>10= NGO health worker (Detail.....................)<br>11= PCSBA<br>12= CHCP<br>13= FWA/HA<br>14= Homeo doctor<br>88= Others (Detail.....................)<br>99= Do not know/Can't remember	01 = Weight<br>02 = Check anemia<br>03 = Check blood pressure<br>04 = Check Edima<br>05 = Check body tempareture<br>06 = Check breast<br>07 = Check lower abdomin<br>08 = Hemorage<br>09 = Foul smelling discharge<br>10= Urine test<br>11 = Provide advise<br>88= Others (Detail.......)

545	What are the complications did you experience within 42 days in your last delivery?<br>[Probe for multiple answer]	01 = Excessive bleeding /Hemorrhage<br>02 = Convulsion/ fits<br>03 = High fever<br>04 = Foul smelling discharge from vagina<br>05 = Wound infections<br>06 = Inverted nipples<br>07 = Engorged breast<br>08 = Tetanus<br>09 = Severe lower abdominal pain<br>10 = Severe chest pain and rapid breathing<br>11 = Uterine prolapse<br>88 = Other (Specify)---------------<br>55 = Didn't face any complication		If no complication then go to 554

No.	Question	Responses	Code	Direction
546	To which provider did you/your family member went/contacted first for those complications?	01= Trditional Birth Attendant<br>02= Relatives/Neighbors<br>03= Trained Traditional Birth Attendant (Govt.)<br>04= Trained Traditional Birth Attendant (Non govt.)<br>05= SACMO/MA (Govt.)<br>06= Nurse/ Midwife/FWV (Govt.)<br>07= Nurse/ Midwife/FWV (Non-govt.)<br>08= Qualified doctor (MBBS)<br>09= VD/Pharmacist<br>10= NGO health worker (Detail.....................)<br>11= PCSBA<br>12= CHW<br>13= CmSS members<br>14= Up members<br>15= CHCP<br>16= FWA/HA<br>17= Homeo doctor<br>18= Didn't contact with anybody<br>88= Others (Detail.....................)<br>99= Do not know		If ans is didn't contact with anybody then go to 554
547	Did the provider refer you?	1 = Yes<br>2 = No		If don't refer then go 553
548	Where did the provider refer you?	01 = At home<br>02= Community Clinic (CC)<br>03= Rural Dispensary (RD)<br>04= Family Welfare Center (FWC)<br>05= Union Health & Family Welfare Center (UH&FWC)<br>06= Upazila Health Complex (UHC)<br>07= Maternal and Child Welfare Center (MCWC)<br>08= District Hospital (DH)<br>09= Medical College Hospital (MCH)<br>10 = Private Hospital (Non profitable/NGO)<br>11 = Private Hospital/Clinic (Profitable)<br>12 = Doctor's private chamber<br>13 = To PCSBA<br>88= Others (Detail.....................)<br>99= Don't know		
549	Did you go the refer place for taking treatment?	1 = Yes<br>2 = No		If don't go refered place then go 553
550	At last, from where did you take the treatment?	01 = At home<br>02= Community Clinic (CC)<br>03= Rural Dispensary (RD)<br>04= Family Welfare Center (FWC)<br>05= Union Health & Family Welfare Center (UH&FWC)<br>06= Upazila Health Complex (UHC)<br>07= Maternal and Child Welfare Center (MCWC)<br>08= District Hospital (DH)<br>09= Medical College Hospital (MCH)<br>10 = Private Hospital (Non profitable/NGO)<br>11 = Private Hospital/Clinic (Profitable)<br>12 = Doctor's private chamber<br>13 = To PCSBA<br>88= Others (Detail.....................)<br>99= Don't know		

551	If you are being referred, during refer who helped you in different stages?<br><br>[Probe for multiple answer]	01= Trditional Birth Attendant<br>02= Relatives/Neighbors<br>03= Trained Traditional Birth Attendant (Govt.)<br>04= Trained Traditional Birth Attendant (Non govt.)<br>05= SACMO/MA (Govt.)<br>06= Nurse/ Midwife/FWV (Govt.)<br>07= Nurse/ Midwife/FWV (Non-govt.)<br>08= Qualified doctor (MBBS)<br>09= VD/Pharmacist<br>10= NGO health worker (Detail.....................)<br>11= PCSBA<br>12= CHW<br>13= CmSS members<br>14= Up members<br>15= CHCP<br>16= FWA/HA<br>17= Homeo doctor<br>88= Others (Detail.....................)<br>99= Do not know		
552	During refer, how did they/he/she help you?<br><br>[Probe for multiple answer]	01= To select the facility and service provider<br>02= Arranged transport to go to health facility<br>03= Took me to the health facility<br>04= Helped to take treatment from the facility<br>05= To communicate with referral facility<br>06= To give money<br>88= Others (Details---------------------------)<br>99= Don't know		
553	During postnatal period, total how much money did you have to spend for postnatal complication?	---------------Tk.		
554	Did you receive iron/iron folic acid tablet in your last pregnancy?	1 = Yes<br>2 = No		If no then go 557
555	How many days did you receive iron/iron folic acid tablet in your last pregnancy?	----------------------Days<br>99 = Don't know/Can't remember		
556	How many tablets did you take in one day in your last pregnancy?	-----------Nos.<br>99 = Don't know/Can't remember		
557	Did you take iron syrup in your last pregnancy?	1 = Yes<br>2 = No		If 554 and 557 ans is no then go 561<br><br>If 554 or 557 anyone ans is yes then ask 560
558	How many days did you take iron syrup in your last pregnancy?	----------------------Days<br>99 = Don't know/Can't remember		
559	How many bottle iron syrup did you take in your last pregnancy?	----------------------bottle syrup<br>99 = Don't know/Can't remember		

No.	Question	Responses	Code	Direction
560	From whom did you get the iron/iron folic acid tablet or syrup?<br><br>[Probe for multiple answer]	01= Trditional Birth Attendant<br>02= Relatives/Neighbors<br>03= Trained Traditional Birth Attendant (Govt.)<br>04= Trained Traditional Birth Attendant (Non govt.)<br>05= SACMO/MA (Govt.)<br>06= Nurse/ Midwife/FWV (Govt.)<br>07= Nurse/ Midwife/FWV (Non-govt.)<br>08= Qualified doctor (MBBS)<br>09= VD/Pharmacist<br>10= NGO health worker (Detail.....................)<br>11= PCSBA<br>12= CHW<br>13= CmSS members<br>14= Up members<br>15= CHCP<br>16= FWA/HA<br>88= Others (Detail.....................)<br>99= Do not know		
561	Did you suffer from any complications or still suffering for long time due to the last pregnancy and delivery?	1 = Yes<br>2 = No<br>9 = Don't know		If no skip to the next section
562	If yes, what were the complications you suffered from or suffering currently?	01 = Perineal tear<br>02 = Urinary incontinence<br>03 = Fistula<br>04 = Genital/Uterine prolapse<br>05 = Dyspareunia<br>06 = Pelvic inflammatory disease<br>07 = Post partum depression<br>08 = External Haemorrhoids<br>09 = Breast problems<br>88= Others (Details--------------------------------)		
563	Did you receive or currently receiving any treatment for those complications?	1 = Yes<br>2 = No<br>9 = Don't know		If no skip to the next section
564	If yes, have you cured completely?	1 = Yes<br>2 = No<br>9 = Don't know		

Section-6: Newborn and child care related practice questions

No.	Question	Responses	Code	Direction
In this part, I would like to ask you some questions regarding newborn and child health care				
601	In case of home delivery, what was used to cut the umbilical cord of the baby?	1 = Blade of delivery kits<br>2 = Blade from other sources<br>3 = Bamboo blade<br>4 = Scissors<br>5 = Didn't cut the umbilical cord<br>7 = Not applicable<br>8= Others (Details.......................................)<br>9 = Don't know		Please circle "Not applicable" code in case of Question no-601 and 602 and move to question no 603
602	Was the instrument boiled before befor cutting the umbilical cord?	1 = Yes<br>2 = No<br>7 = Not applicable<br>9 = Don't know		

No.	Question	Responses	Code	Direction
603	What was used on navel after the delivery?<br><br>(Pobe for more answers)	01 = Antibiotic powder/oientmet<br>02 = Detol/Savlon/Hexisol (Blue colored)<br>03 = Clorohexidine (Red colored)<br>04 = Jenshion Voilet (Blue colored ink)<br>05 = Spirit/Alchole<br>06 = Borik powder<br>07 = Telkom powder<br>08= Mustard oil with garlic<br>09 = Coconut oil<br>10 = Cucumbar juice<br>11 = Ginger juice<br>12= Cinnabar<br>13 = Ash<br>14 = Chewing rice/rice<br>15 = Didn't use anything<br>88 = Others (Details.......................................)<br>99 = Don't know		
604	When was the body of the newborn wiped immediately after birth?	Minute: ☐☐<br>98 = Didn't dry<br>99 = Don't know		
605	When was the body of the newborn wrapped immediately after birth?	Minute: ☐☐<br>98 = Didn't dry<br>99 = Don't know		
606	After the birth, did you measure the weight of your baby?	1 = Yes<br>2 = No<br>9 = Don't know		Please go to 608 if answer is eithr "No" or "Don't know"
607	If yes, what was the weight of your baby at the time of delivery?	---------------------- Gram<br>9999 = Don't know		
608	When did you initiate breastfeeding your baby immediately after birth?	a. = Minute ☐☐<br>b. = Hour ☐☐<br>c. = Day ☐☐<br>98 = Didn't initiate<br>99 = Don't know		
609	Did you give colostrums to your baby immediately after birth?	1 = Yes<br>2 = No		
610	When did you breastfeed your baby after birth?	a. = Minute ☐☐<br>b. = Hour ☐☐<br>c. = Day ☐☐<br>98 = Didn't iniciate<br>99 = Don't know		
611	Did you feed anything to your child other than breastmilk withing 3 days of birth?	1 = Yes<br>2 = No Please move to 613 if anser is **"No"**		Please move to 613 if anser is "No"
612	If yes, what wwa given to the newborn baby?<br><br>(Pobe for more asnswers)	01 = Milk (except breast milk)<br>02 = Only water<br>03 = Sugar or glucose water<br>04 = Gripe water<br>05 = Water with sugar and solt<br>06 = Juice of fruits<br>07 = Baby mixed<br>08 = Tea/Saline<br>09 = Honey<br>88 = Other (Specify..)		

No.	Question	Responses	Code	Direction
613	How long after delivery the baby was (Name) bathed for the first time?<br><br>[Please write in hour if answer is less than one day and write in day if answer is less than a week]	a. = Day ☐☐<br>b. = Hour ☐☐<br>c. = Minute ☐☐<br>98 = Didn't bath yet<br>99 = Don't know		
614	When was the hair of the baby shaved first?	a. = Month ☐☐<br>b. = Day ☐☐<br>c. = Hour ☐☐<br>98 = Didn't shave the hair yet<br>99 = Don't know		
615	During the first month (28 days) of life, did (Name) the child have any complications?<br><br>[Probe for multiple answer]	01 = Weak cry<br>02 = Absent cry<br>03 = Unable to suck or breast feed<br>04 = Lethargy<br>05 = Cold hands and feet<br>06 = Rapid breathing<br>07 = Chest indrawing<br>08 = High temparature (fever)<br>09 = Convulsion/unconsciousness<br>10 = Umbilical discharge<br>11 = More than 10 skin pustules<br>12 = Red eyes with discharge<br>13 = Jaundice for more than 14 days<br>88 = Other ------------------------------<br>55 = Didn't have any complication		**If didn't have any complication skip to 624**
616	To which provider did you/your family member went/contacted first for those complications?	01= Trditional Birth Attendant<br>02= Relatives/Neighbors<br>03= Trained Traditional Birth Attendant (Govt.)<br>04= Trained Traditional Birth Attendant (Non govt.)<br>05= SACMO/MA (Govt.)<br>06= Nurse/ Midwife/FWV (Govt.)<br>07= Nurse/ Midwife/FWV (Non-govt.)<br>08= Qualified doctor (MBBS)<br>09= VD/Pharmacist<br>10= NGO health worker (Detail.....................)<br>11= PCSBA<br>12= CHW<br>13= CmSS members<br>14= Up members<br>15= CHCP<br>16= FWA/HA<br>17= Homeo doctor<br>18= Didn't contact with anybody<br>88= Others (Detail.....................)<br>99= Do not know		If ans is didn't contact with anybody then go to 624

No.	Question	Responses	Code	Direction
617	Did the provider refer you?	1 = Yes<br>2 = No		If don't refer then go 623
618	Where did the provider refer you?	01 = At home<br>02= Community Clinic (CC)<br>03= Rural Dispensary (RD)<br>04= Family Welfare Center (FWC)<br>05= Union Health & Family Welfare Center (UH&FWC)<br>06= Upazila Health Complex (UHC)<br>07= Maternal and Child Welfare Center (MCWC)<br>08= District Hospital (DH)<br>09= Medical College Hospital (MCH)<br>10 = Private Hospital (Non profitable/NGO)<br>11 = Private Hospital/Clinic (Profitable)<br>12 = Doctor's private chamber<br>13 = To PCSBA<br>88= Others (Detail.....................)<br>99= Don't know		
619	Did you go the refered place for taking treatment?	1 = Yes<br>2 = No		If don't go refered place then go 623
620	At last, from where did you take the treatment?	01 = At home<br>02= Community Clinic (CC)<br>03= Rural Dispensary (RD)<br>04= Family Welfare Center (FWC)<br>05= Union Health & Family Welfare Center (UH&FWC)<br>06= Upazila Health Complex (UHC)<br>07= Maternal and Child Welfare Center (MCWC)<br>08= District Hospital (DH)<br>09= Medical College Hospital (MCH)<br>10 = Private Hospital (Non profitable/NGO)<br>11 = Private Hospital/Clinic (Profitable)<br>12 = Doctor's private chamber<br>13 = To PCSBA<br>88= Others (Detail.....................)<br>99= Don't know		
621	If you are referred, during refer, who helped you in different stages?<br><br>[Probe for multiple answer]	01= Trditional Birth Attendant<br>02= Relatives/Neighbors<br>03= Trained Traditional Birth Attendant (Govt.)<br>04= Trained Traditional Birth Attendant (Non govt.)<br>05= SACMO/MA (Govt.)<br>06= Nurse/ Midwife/FWV (Govt.)<br>07= Nurse/ Midwife/FWV (Non-govt.)<br>08= Qualified doctor (MBBS)<br>09= VD/Pharmacist<br>10= NGO health worker (Detail.....................)<br>11= PCSBA<br>12= CHW<br>13= CmSS members<br>14= Up members<br>15= CHCP<br>16= FWA/HA<br>17= Homeo doctor<br>88= Others (Detail.....................)<br>99= Do not know		

No.	Question	Responses	Code	Direction
622	During refer, how did they/he/she help you?<br><br>[Probe for multiple answer]	01= To select the facility and service provider<br>02= Arranged transport to go to health facility<br>03= Took me to the health facility<br>04= Helped to take treatment from the facility<br>05= To communicate with referral facility<br>06= To give money<br>88= Others (Details---------------------------)<br>99= Don't know		
623	How much money did you have to spend for newborn health complications?	---------------Tk.		
**Child Care**				
624	Are you still breastfeeding (Name)?<br>CHECK-CHILD ALIVE?	1 = Yes<br>2 = No<br>7 = Not applicable		
625	For how many months did you exclusively breastfeed the baby (Name)?	Months<br><br>99= Don't know		
626	When did you give an extra food to your baby after the child birth (liquid or solid or semi solid food except breast milk)	------------ days<br>------------- months<br>98= Yet to start<br>99= Don't know		If didn't start to give liquid or solid or semi solid food yet then skip to 628
627	How many times did you give solid or semi solid food except liquid food to your baby in last 24 hours?	------------- Times<br>99= Don't know/Can't remember		
628	Do you measure your baby's weight (GMP) regularly?	1 = Yes<br>2 = No		
629	Have you received any card to keep record of each weight measured?	1 = Yes<br>2 = No		If no skip to 631
630	If yes, who provided this card?	01= Trditional Birth Attendant<br>02= Relatives/Neighbors<br>03= Trained Traditional Birth Attendant (Govt.)<br>04= Trained Traditional Birth Attendant (Non govt.)<br>05= SACMO/MA (Govt.)<br>06= Nurse/ Midwife/FWV (Govt.)<br>07= Nurse/ Midwife/FWV (Non-govt.)<br>08= Qualified doctor (MBBS)<br>09= VD/Pharmacist<br>10= NGO health worker (Detail.....................)<br>11= PCSBA<br>12= CHW<br>13= CmSS members<br>14= Up members<br>15= CHCP<br>16= FWA/HA<br>88= Others (Detail.....................)<br>99= Do not know		
631	Have you got the moni mix/micro nutrient powder for your last baby?	1 = Yes<br>2 = No		If no skip to 633

No.	Question	Responses	Code	Direction
632	If yes, from whom did you get this?	01 = NGO health worker (Details-----------------)<br>02= PCSBA<br>03 = CHW<br>04= CmSS members<br>05= UP members<br>06= CHCP<br>07= FWA/HA<br>88= Others (Detail.....................)<br>99= Do not know		
633	Did your baby (Name) receive any vaccination to prevent him/her from getting diseases?	1 = Yes<br>2 = No		If no skip to next section
634	From where did you get this vaccination?	01 = EPI center<br>02= Sattelite<br>03= Special Center<br>04= Special Camp<br>05= Community Clinic (CC)<br>06= Rural Dispensary (RD)<br>07= Family Welfare Center (FWC)<br>08= Union Health & Family Welfare Center (UH&FWC)<br>09= Upazila Health Complex (UHC)<br>10 = Maternal and Child Welfare Center (MCWC)<br>11 = District Hospital (DH)<br>12 = Medical College Hospital (MCH)<br>88= Others (Detail.....................)<br>99= Do not know		

Section-7: Familiy planning related practice questions

Family planning				
701	If you are not currently pregnant, which family planning methods you or your husband is using right now?	01= Female sterilization<br>02 = Male sterilization<br>03 = Pill<br>04 = IUD<br>05 = Injectables<br>06 = Implant/Norplant<br>07 = Condom<br>08 = Safe period<br>09 = Withdrawl<br>10 = None<br>11 = Currently pregnant<br>88= Other (specify --------------------------------)		Checked currently pregnant or not if yes then circle currently pregnant<br><br>and if use nothing then skip to next section
702	From where or whom did you get those family planning methods?<br><br>[Probe for multiple answer]	01= Trditional Birth Attendant<br>02= Relatives/Neighbors<br>03= Trained Traditional Birth Attendant (Govt.)<br>04= Trained Traditional Birth Attendant (Non govt.)<br>05= SACMO/MA (Govt.)<br>06= Nurse/ Midwife/FWV (Govt.)<br>07= Nurse/ Midwife/FWV (Non-govt.)<br>08= Qualified doctor (MBBS)<br>09= VD/Pharmacist<br>10= NGO health worker (Detail.....................)<br>11= PCSBA<br>12= CHCP<br>13= FWA/HA<br>88= Others (Detail.....................)<br>99= Do not know		

Section-8: Your role in decision making and family cooperation

No.	Question	Responses	Code	Direction
In this section, I will ask you some questions related to decision making regarding health care seeing and other issues and family cooperation				
801	Who is the main decision maker to seek heralth care in case of your illness?	01 = Herself<br>02 = Husband<br>03 = Both husband and herself<br>04 = Father in law<br>05 = Mother in law<br>06 = Mother<br>07 = Father<br>08 = Brothe in law/ sister in law<br>88 = Others (Details -----------------------------)		
802	Who is the main decision maker to seek heralth care in case of your child's illness?	01 = Herself<br>02 = Husband<br>03 = Both husband and herself<br>04 = Father in law<br>05 = Mother in law<br>06 = Mother<br>07 = Father<br>08 = Brothe in law/ sister in law<br>88 = Others (Details -----------------------------)		
803	Who is the main decision maker to purchase daily needs/commodities of your household?	01 = Herself<br>02 = Husband<br>03 = Both husband and herself<br>04 = Father in law<br>05 = Mother in law<br>06 = Mother<br>07 = Father<br>08 = Brothe in law/ sister in law<br>88 = Others (Details -----------------------------)		
804	Who is the main decision maker in case of visiting relitives' household?	01 = Herself<br>02 = Husband<br>03 = Both husband and herself<br>04 = Father in law<br>05 = Mother in law<br>06 = Mother<br>07 = Father<br>08 = Brothe in law/ sister in law<br>88 = Others (Details -----------------------------)		
805	Could you go alone to service center if you need to seek health care or do you take small child with you?	1= Go alone<br>2 = Take small child with her<br>3 = Cannot go alone		
806	Could you go alone to any service center if you child's need to seek health care or do you take small child with you?	1= Go alone<br>2 = Take small child with her<br>3 = Cannot go alone		
807	Are you allowed to go alnoe if it needs to visit any relative's household?	1= Yes<br>2 = No		

Section-09: Community Mobilizaion (CmSS, Folk song, etc related questions)

No.	Question	Responses	Code	Direction
901	According to you, is there any group (community clinic group or care support group) in your area working to improve maternal and child health?	1 = Yes u<br>2 = No<br>99 = Do not know		If no or do not know, go to 904
902	Did you receive any help from these community clinic group or CARE support group in your last pregnancy?	1 = Yes u<br>2 = No		If no go to 904
903	If received, what kind of help?<br><br>[Probe for multiple answer]	01 =provided information about health facilities<br>02 = Linked with the health service providers<br>03=Took to the health facilitity<br>04= Helped in taking treatment from health facility<br>05= Arranged transport to go to the health facility<br>06= provided economic support to receve health services<br>07= Discussed about birth planning<br>08= Linked with PCSBA<br>88 = Others (Specify---------------------------------)		
904	Did you take part in any folk song/ street theatre arranged by CARE Bangladesh?	1 = Yes u<br>2 = No		If no go to 1001
905	If so, what did you learn from those?<br><br>[Probe for multiple answer]	01=About PCSBA services<br>02= pregnancy complications ©<br>03=Child Health complications<br>04=Need for care during pregnancy<br>05= Need for delivery care with skilled birth attendant<br>06= Need for post delivery care<br>07= Nutrition<br>08= Referral<br>09= Negative effects of early marriage<br>10= Family planning<br>11=CmSS activities<br>88= Others (Specify----------------------------------)		

Section-10: Entrepreneurship, Health Care cost and Health financing

No.	Question	Responses	Code	Direction
1001	Which services did you or yor family receive from PCSBA?<br><br>[Probe for multiple answer]	01=ANC<br>02= DC<br>03=PNC<br>04= Child Care<br>05= Nutrition related services<br>06= Referral services<br>07= Health related information and suggestion<br>08= Never communicate with PCSBA / don't know about PCSBA<br>88 = others(---)<br>98= Respondent and her family did not receive any services from PCSBA		If the answer is08 orv 99 then go to Q1008
1002	In case of refer which sorts of assistance/supports did you or your family get from PCSBA?<br><br>[Probe for multiple answer]	1= helped to take decision in selecting service provider<br>2= Accompanied to service center<br>3= Assisted with money.<br>4= Managed DSF (Demand Side Financing)<br>5= Managed vehicle to go to service center<br>6= Helped to communicate with referral health care center<br>9= Dd not need to refer/ never referred		

No.	Question	Responses	Code	Direction
1003	How do you assess the service quality of PCSBA? (Prob: Skills in service providing, behavior, helping in refer etc.)	1= Very good<br>2= Good<br>3= mediocre/ average<br>4= Bad<br>5= Very bad<br>6= Did not receive any services from PCSBA<br>8 = Others (--)		If answer is 1 or 2 then go to Q.1005 and do not ask Q 1006<br><br>If answer 3, 4 or 5 then go to Q1006 and avoid Q 1005<br><br>If the answer is 6 then do not ask the Q1005, 1006
1004	If the service you have received from PCSBA is good or very good then what are the reasons?<br><br>[Probe for multiple answer]	01= Provide service in home/ door to door<br>02= Skilled<br>03= Available<br>04= Good behavior<br>05= Known person<br>06= Listen carefully about the problems and try to solve it<br>07= Takes reasonable amount of taka/ takes a lesser amount of money<br>08= Provides good advbice<br>88 = others(--)		
1005	If the service you have received from PCSBA is not good then what are the reasons?<br>[Probe for multiple answer]	1= Did not come home<br>2= Unskilled<br>3= Not available in crisis situation<br>4= Bad behavior<br>5= Did not listen to the problems carefully<br>6= Alwayes demands money<br>7= Costly<br>8 = Others(--)		
1006	Did you or your family get any donation or money from the CMSS or UP or from any other members of your society to receive service from PCSBA?	1 = Yes<br>2 = No<br>6= Did not receive any services from PCSBA<br>9 = Don’t know		
1007	Did the CMSS or UP or any other person of your society donate money to PCSBA to provide service to you or your family?	1 = Yes<br>2 = No<br>6 = Did not receive any services from PCSBA<br>9 = Don’t know		
1008	What is your opinion about the amount which PCSBA takes after providing services?	1= Takes much/costly<br>2= Reasonable<br>3= Less<br>4= No opinion<br>6= Did not receive any services from PCSBA<br>8 =Others(--)		
1009	Did you or your family buy any goods from PCSBA except health service?<br><br>[Probe for multiple answer]	1= Regular medicines<br>2= Nutritional elements<br>3= Family planning materials<br>4= Sanitary napkins<br>8 = Others(--)<br>9= Did not buy anything		

No.	Question	Responses	Code	Direction
1010	How did your family pay the cost of the services (ANC, DC, PNC) which you had received in your last pregnancy?<br><br>[Probe for multiple answer]	01= From income<br>02= From savings<br>03= With the help of family<br>04= With the help of others/ members outside of the family<br>05= By borrowing money from any person of the family<br>06= By borrowing money from any person out side of the family<br>07= By taking loan from microcredit organization<br>08= By selling any unprofitable goods of the family<br>09= By selling any profitable goods/properties of the family<br>10=FromDSF or government schem(DSF)<br>88 =Others..<br>99= Don't know		
1011	Do you or your family members save money for receiving health services?	1 = Yes<br>2 = No<br>9 = Don't know		If yes then go to the another section
1012	Do you or your family have interest in saving money regarding health care?	1 = Yes<br>2 = No<br>9 = Don't know		If no or don't know then go to Q 1014
1013	If interested, then how much money are you or your family members interested to save in a month (taking consideration of others expenditure in your family)?	.......................taka		
1014	Do you have the ability to save the money?	1 = Yes<br>2 = No<br>9 = Don't know		

Section: 11: Non communicable disease related information of the HH members aged 35 years and above

No.	Question	Responses	Code	Direction
1101	What are the ways to prevent Non comunicable diseases such as: Diabetes, Heart diseases, High blood pressure, Cancer, etc?<br><br>[Probe for multiple answer]	01 = Controlling weight<br>02= Eating lot of vegetables<br>03= Taking adequate salt<br>04= Avoiding Tobacco and related stuff<br>05= Being stress free<br>06= Doing regular physical labour<br>07 = Walking regularly<br>08= Avoiding excess oil and fatty food/ fast food etc.<br>09= Taking adequate rest<br>10= Ckecking blood pressure regularly<br>11= Ckecking diabetis regularly<br>88 = Others (Specify---------------------------------)<br>99 = Do not know		

1102	Questions related to blood pressure			Questions related to diabetis			Questions related to Tobacco use	
Write the Name of people above 35 years	a)Did he/she ever check blood pressure<br>1=Yes<br>2 =No<br>9=Do not know	b) Did he /she have blood pressure checked by doctor or nurses?<br>1=Yes<br>2 =No<br>7=Not applicable	c) Does he/ she take any medicine to control high blood pressure<br>1=Yes<br>2 =No<br>7=Not applicable	d) Did ever doctor or nurse check his or her diabetis to recognize diabetis<br>1=Yes<br>2 =No<br>7=Not applicable	e) Does he/ she take any medicine to control diabetis<br>1=Yes<br>2 =No<br>7=Not applicable	f) How is she/he taking medicines<br>1=Injection<br>2 =Orally<br>3 = Orally and injection<br>7= Not applicable	g)Does he take Tobacco related stuff regularly<br>1=Yes<br>2 =No<br>Don't not know	h)If yes, what kind of Tobacco stuff do you use?<br>(1=Bidi/ Cigarate<br>2 = Jorda/ Tobacco,<br>3 = Gul 7= Not applicable
1102a.								
1102b.								
1102c.								
1102d.								
1102e.								

1103	Who provided the above information (multiple answers accepted)	01=Respondent herself<br>02 =Other family members<br>03 = People above 35 years old		

Section-12: Mass media exposure, cell phone use and NGO/micro-credit involvement

No.	Question	Responses	Code	Direction
1201	Do you read newspaper or magazine?	1= Yes<br>2 = No		If no, skip to 1203
1202	How often do you read newspaper or magazine?	1 = Almost every day<br>2 = At least once a week<br>3 = Less than once a week		
1203	Do you watch television?	1= Yes<br>2 = No		If no, skip to 1205
1204	How often do you watch television?	1 = Almost every day<br>2 = At least once a week<br>3 = Less than once a week		
1205	Are you involved with any micre-credit or community based organization (CBO) organization?	1= Yes<br>2 = No		If no, skip to 1207
1206	What are the organizations that you are involved in?	1 = Grameen Bnak<br>2 = BRAC<br>3 = BRDB<br>4 = ASHA<br>5 = Proshika<br>6 = CBO (----------)<br>8 = Other (specify---)		CBO such as Village Development Committee, Clubs etc
**Utility of Cell phone use in healthcare seeking**				
1207	Does your family have own mobile phone?	1 = Yes<br>2 = No		If no then go directly 1210
1208	Do you have own any mobile phone?	1 = Yes<br>2 = No		
1209	Does anyone in your family members/Do you have own smart phone?	1 = Yes<br>2 = No		If needed observe or if you have smartphone then show and ask
1210	Do you have 24 hours access to a mobile phone?	1 = Yes<br>2 = No		

No.	Question	Responses	Code	Direction
1211	Can you read the SMS in the mobile?	1 = Yes<br>2 = No		If no skip to 1213
1212	If yes, then which language do you read SMS?	1 = Bangla<br>2 = English<br>3 = Both		
1213	Have you ever communicated with anyone for healethcare purpose?	1 = Yes<br>2 = No		If no then end the interview
1214	If yes, with whom did you communicate?<br><br>[Probe for multiple answer]	1= PCSBA<br>2 = Relatives<br>3= CHW<br>4 = CmSS members<br>5 = Health worker (others)<br>6= Doctor<br>8 = Other (specify---)		
1215	For what purposes did you communicate?<br><br>[Probe for multiple answer]	1 = Pregnancy complication<br>2 = Delivery complication<br>3 = Postnatal complication<br>4= Newborn complication<br>5= Child health problem<br>6= General health problem<br>8 = Other (specify---)		
1216	Did they respond to your call?	1 = Yes<br>2 = No		If no then end the interview
1217	Do you think that the phone call was useful in that situation?	1 = Yes<br>2 = No		

Thank you for taking part in the interveiw and your time

Ending time of data collection: ☐☐ ☐☐ Hour Minute

Date: ☐☐ ☐☐ ☐☐☐☐ Day Month Year

How much time was needed to complete the interview: ☐☐ ☐☐ Hour Minute

For Official Use only

A	Name of the area			
B	Result Status of the interview	1 = Complete<br>2 = Incomplete<br>3 = Refusal		
C	Number of attempts were needed to complete the interview	1 = Once<br>2 = Twice<br>3 = More than twice		
D	Data collector’s name and Signature:<br>Date: -------/------/2016			
E	Supervisor’s name and Signature:<br>Date: -------/------/2016			
F	Research investigator’s name and Signature:<br>Date: -------/------/2016			
	Data entry person’s name and Signature:<br>Date: -------/------/2016			

Field Note
