# Supplementary material for: Filling the human resource gap through public-private partnership: Can private, community-based skilled birth attendants improve maternal health service utilization and health outcomes in a remote region of Bangladesh?
Source: PLoS One. 2020 Jan 17;15(1):e0226923. doi: 10.1371/journal.pone.0226923 (PMC6968857; doi:10.1371/journal.pone.0226923)
Supplement: S2 File — (PDF) [file pone.0226923.s002.pdf]

Consent Form (Survey with Mother) [In English]

Protocol No. PR-12083
Protocol Title: Baseline study on GSK supported Community Health Worker (CHW) initiative
Principal Investigator: Bidhan Krishna Sarker
Organization: International Centre for Diarrhoeal Disease Research, Bangladesh (icddr,b)

Purpose of the research

Background:
I/ We have (please tell your name) come from icddr,b (Cholera Hospital at Dhaka). We are currently working with CARE and conducting a baseline study to explore the situation of maternal, neonatal and child health, family planning (MNCH&FP) and nutrition in your area. You may know that, in our country maternal, infant and child mortality rates are still high compared to other countries. In order to reduce abovementioned mortality rates, CARE has initiated the Community Health Worker (CHW) program in this area and for effective implementation of this program; we need baseline information on current knowledge and practices about MNCH&FP and Nutrition. The findings from the research will be used to monitor and evaluate the future CARE intervention.

Why invited to participate in the study?
You have been selected as a participant for the study because you live in this area where CARE will implement an intervention or any other organization may implement projects and you are the primary target audience which connected you directly to this survey. Your active participation and in-depth knowledge share will enrich our current study and future direction.

What is expected from the participants of the research study?
I would like to invite you to participate in a survey, which will take about one hour. You will be asked questions about MNCH&FP and Nutrition related knowledge and practice. I assure you that the information you provide will only be utilized for this study.

Risk and benefits
By participating in this study, you will not be subjected to any potential risk. We are unable to pay you any money for your participation.

Privacy, anonymity and confidentiality
You can be assured that your participation and all information given by you will be kept strictly confidential and anonymous. I need your kind cooperation and free and honest discussions. Please be sure that there will be no need and no chance to identify you after this interview. The data will be kept under the confidential and strict supervision of the head of the project under lock and key. None other than the investigators of this research would have an access to the information. Any personal information related to identity of the informant would be kept completely secret. You would be able to talk to any of our project staff if you want and we are obliged to answer any questions.

Future use of information
In future, the findings of this project will be utilized for scientific knowledge generating activities and other researchers would have access to findings of this study. However, the privacy, anonymity and confidentiality of information will be maintained in such a way that participants in no way could be linked.

Right not to participate and withdraw
Your participation in the interview is completely voluntary. It is you and only you who will decide whether you participate in this study. You can stop any time without any obligation during our discussion if you feel uncomfortable. Refusal to take part in or withdrawal from the interview will involve no penalty and no loss of your services. We can go to any private place according to your choice or if you agree, I can make arrangements so that we can sit and freely talk with privacy. You can talk in your free time, as I have no time to pressure. In case of any need, we may sit again if you permit.

We will happily provide you further information about the study, if any, now or at a later time. You may communicate with the principal investigators of the study or her/his designated person at the contact address given below. We will answer to your question related to this study. If you kindly give me your consent, then and only then I can begin the interview.

If you agree to our proposal of enrolling you in our study, please indicate that by putting your signature or your left thumb impression at the specified space below

Thank you for your cooperation

______________________________________ ____________________
Signature or left thumb impression of participant Date

_____________________________________ ____________________
Signature or left thumb impression of Date
Parent/ Guardian/ Attendant

______________________________________ ____________________
Signature or left thumb impression of the witness Date

______________________________________ ___________________
Signature of the PI or his/her representative Date

(NOTE: In case of representative of the PI, she/he shall put her/his full name and designation and then sign)
Please contact here for further queries:

Bidhan Krishna Sarker<br>Centre for Reproductive Health<br>icddr,b<br>Mohakhali, Dhaka 1212,<br>Phone: 880-2-886 0523-32 Ext.2265<br>E-mail: bidhan@icddrb.org	M A Salam Khan<br>IRB Secretariat<br>icddr,b<br>Mohakhali, Dhaka 1212,<br>Phone: 2- 988-6498 PABX 8860523-32 Extension. 3206<br>E-mail: salamk@icddrb.org

Survey questionnaire for 'Baseline study on GSK supported Community Health Worker (CHW) initiative'

Starting time of data collection: [ ][ ] Hour [ ][ ] Mins

Date of data collection: Date: [ ][ ] Day [ ][ ] Month [ ][ ] Year

ID number: [ ][ ][ ][ ]

Screening questions:

A	When did your last childbirth take place?	1 = 1-12 months<br>2 = 13-59 months ago<br>3 = More than 59 months ago<br>4 = Less than 1 month ago	

[IF THE RESPONSES ARE GIVEN (1 OR 2) THEN START THE INTERVIEW OTHER WISE GO TO THE NEXT RESPONDENT]

Section-1: Respondent's identity:

No.	Question	Responses	Code	Direction
101	Name of the respondent			Try to collect
102	Name of husband			
103	Household number			
104	Name of village/mouza	--		Use code if as per study plan
105	Name of Union	--		
106	Name of sub-district	-----------------------------------		
107	Name of district	----------------------------------		
108	Type of respondent	1= Respondent is a mother having childbirth 1-11 months ago<br>2= Respondent is a mother having childbirth 12-59 months ago<br>3= Respondent is a mother having childbirths both 1-11 month and 12-59 months ago.		
**Contact number and location in detail:**				
109	Contact number (cell or telephone):			
110	Contact person of the given number:			
111	Relationship of contact person with respondent			
112	Detail location of the household with landmark:			

Household level family member's information

No.	Name of regular family member	*Relation with respondent	Sex; 1= male 2= female	Age in month & years	**Marital status	Is currently pregnant 1=Y, 2=N, 3=N/A	Does have U-5 child 1=Y, 2=N, 3=N/A	Education Class (passed) 99 =N/A	***Type of schooling	Occupation
113										
114										
115										
116										
117										
118										
119										
120										
121										
122										
123										

Coding

*Relation with respondent	**Marital Status	Education code:	***Types of schooling	****Occupation
01= Self; 02 = Husband; 03 = Son/Son-in-law; 04 = Daughter/ Daughter-in-law; 05= Nephew/Niece<br>06 = Mother/Mother-in-law; 07 = Father/Father-in-law; 08 = Brother/ brother in law; 09 = Sister/ Sistern in law; 10= Step son or daughter; 11=Other (Specify---------------------------)<br>99 = No relation,<br>N.B: Family members who are living together under same household	1= Currently married<br>2= Widowed<br>3 = Divorced<br>4 = Separated<br>5 = Unmarried<br>9 = Not applicable	99 =Not applicable<br><br>[when age is under 5]	1= Formal<br>2= Informal<br>3= Madrasha<br>4= Don't know	01= Service; 02=Day labor;<br>03= Business;<br>04= Agriculture/Farming/Fisherman<br>05= Handicraft;<br>06= Rickshaw/Van puller;<br>07= Motorized transport worker;<br>08= Boat man; 09 = Student<br>10= Homemaker; 11 = Unemployed<br>12= Child (under 10 years) /Older (above 60 years)<br>13 = Others (specify---------),<br>99 = Not applicable

Section-2: Basic Household characteristics:

No.	Question	Response	Code	Direction
201	What is the main source of drinking water for members of your household?	1 = Supply water<br>2 = Tube well water<br>3 = Dug well<br>4 = Rainwater<br>5 = Pond / River / Canal<br>6 = Bottled water<br>7 = Others (Specify)		
202	Do you boil the water to make it safer to drink?	1= Yes<br>2 = No		
203	What is the main source of water used by your household for other purposes such as cooking and handwashing?	1 = Supply water<br>2 = Tube well water<br>3 = Dug well<br>4 = Rainwater<br>5 = Pond / River / Canal<br>6 = Bottled water/ Mineral water<br>7 = Others (Specify)		
204	What kind of toilet facility do members of your household usually use?	1 = Sanitary latrine (linked with sewarage)<br>2 = Sanitary latrine (with septic tank)<br>3 = Pit latrine (with slab)<br>4 = Pit latrine (without slab)<br>5 = Open pit<br>6 = Bucket toilet<br>7 = Hanging toilet<br>8 = No facility/ Bush/open space/field<br>9 = Others (Specify)------------------------		

No.	Question	Response	Code	Direction
205	Do you share this toilet facility with other household?	1= Yes<br>2 = No<br>3 = Not applicable		
206	Does your household have any of the following items which are functioning?			
	a) Electricity	1 = Yes 2 = No		
	b) Radio/Casset/CD player/DVD player	1 = Yes 2 = No		
	c) Television	1 = Yes 2 = No		
	d) Computer	1 = Yes 2 = No		
	e) Mobile telephone	1 = Yes 2 = No		
	f) Fixed or non-mobile telephone	1 = Yes 2 = No		
	g) Refrigerator	1 = Yes 2 = No		
	h) Almirah/wardrobe/ showcase/ Alna	1 = Yes 2 = No		
	i) Table	1 = Yes 2 = No		
	j) Chair	1 = Yes 2 = No		
	k) Cot	1 = Yes 2 = No		
	l) Watch	1 = Yes 2 = No		
	m) Bicycle	1 = Yes 2 = No		
	n) Motorcycle/motor scooter/tempo	1 = Yes 2 = No		
	o) Animal-drawn cart (cart drawn by horse)	1 = Yes 2 = No		
	p) Car/ truck	1 = Yes 2 = No		
	q) Boat with a motor	1 = Yes 2 = No		
	r) Ricksha/van	1 = Yes 2 = No		
	s) Others (Details...................................)	1 = Yes 2 = No		
207	What type of fuel does your household mainly use for cooking?	01 = Electricity<br>02 = LPG<br>03 = Natural Gas<br>04 = Biogas<br>05 = Kerosene<br>06 = Coal/Lignite<br>07 = Charcoal<br>08 = Wood<br>09 = Straw/Shrubs/grass<br>10 = Agricultural crop<br>11 = Animal dung<br>12= Others (Specify)-----------------		
208	What are the construction materials of different parts of your house?			
	a. Main material of the roof	1 = Pucca (Bricks and Cement)<br>2 = Corrugated iron<br>3 = Tally<br>4 = Straw/Bamboo/Leaves<br>5 = Others (Specify)-------------------		
	b. Main material of the exterior walls	1 = Pucca (Bricks and Cement)<br>2 = Corrugated iron/wood<br>3 = Clay/mud<br>4 = Straw/Bamboo/Leaves<br>5 = Others (Specify)-------------------		
	c. Main material of the floor	1 = Pucca (Bricks and Cement)<br>2 = Semi – Pucca<br>3 = Clay/Kutcha<br>4 = Wood/Bamboo		
209	How many roms in this household are used for sleeping?	--------------------------Nos.		
210	Does your household own any livestock, herds, other farm animals, or poultry?	1= Yes<br>2 = No		If no, skip to 212
211	How many of the following animal does your household own?	a. Cows/buffalo		Write 00' if no animal
		b. Goats/sheep		
		c. Chickens/ducks/pegion		
212	Does your household own any homestead?<br>[Probe: does your household own homestead in any other places?]	1= Yes<br>2 = No		

213	How much land does your household own (other than the homestead land)?	--------------------Decemal		Write 00' if no land

Section-3: Basic individual characteristics and socio-demographic information:

No.	Question	Responses	Code	Direction
301	What is your age?	--------------(Completed Years)		
302	What is your religion?	1 = Islam<br>2 = Hindu<br>3 = Christian<br>4 = Buddhist<br>5 = Other		
303	What is your completed year(s) of education?	------ Completed year(s) of education		
304	What is your husband's completed year of education?	------ (Completed years of education)		
305	What is your current marital status?	1 = Currently married<br>2 = Divorced<br>3 = Separated<br>4 = Widowed<br>5 = Deserted<br>6 = Never married		
306	Are you currently involved in any kind of income generating activities?	1 = Yes<br>2 = No		
307	What is your primary occupation?	01 = Service<br>02 = Day labor<br>03 = Business<br>04 = Agriculture/Farming/Fisherman<br>05 = Handicraft<br>06 = Rickshaw/Van puller<br>07 = Motorized transport worker<br>08 = Boat man<br>09 = Student<br>10 = Homemaker/ housewife<br>11 = Unemployed<br>12 = Others (specify--------------------)		
308	What is your husband's primay occupation?	01 = Service<br>02 = Day labor<br>03 = Business<br>04 = Agriculture/Farming/Fisherman<br>05 = Handicraft<br>06 = Rickshaw/Van puller<br>07 = Motorized transport worker<br>08 = Boat man<br>09 = Student<br>10 = Homemaker<br>11 = Unemployed<br>12 = Others (specify--------------------)<br>99 = Not Applicable (if husband is not alive)		
309	How many members do you have in your family?	-------------------------Nos.		
310	How many of your family members involved in income generation?	-------------------------Nos.		
311	What is your individual average monthly income?	---------------------------------Tk.		Write '00' if no income
312	What is your average family monthly income?	---------------------------------Tk.		Write '00' if no income
313	What is your average family monthly expenditure?	---------------------------------Tk.		

No.	Question	Responses	Code	Direction
314	Who usually make the decision in seeking healthcare from outside home for women or child in your household?<br>(emphasis both mobility and expenditure)	1= You alone<br>2= Husband alone<br>3= You and your husband combinely<br>4= Father in law<br>5= Mother in law<br>6= Mother<br>7= Father<br>8= Brother in law/Sister in law<br>9 = Others (specify--------------)		
315	Who usually make the decision in purchisng daily household needs in your household?	1= You alone<br>2= Husband alone<br>3= You and your husband combinely<br>4= Father in law<br>5= Mother in law<br>6= Mother<br>7= Father<br>8= Brother in law/Sister in law<br>9 = Others (specify--------------)		
**Reproductive information**				
316	At what age were you married for the first time?	Age in completed years		
317	How many children have you ever given birth to?	---------------------		
318	How many living children do you have now?	--------------		
319	What was the date of your last delivery?	-------- --------- ---------<br>Day Month Year<br>Can't say-----------------99		
320	What was the mode of your last delivery	1= Normal delivery<br>2= Caesarean delivery<br>3= Others (sprcify----------------)		
321	Are you pregnant now?	1 = Yes<br>2 = No		If no, skip to q. 323
322	How many months pregnant you are?<br>[RECORD NUMBER OF COMPLETED MONTHS]	Months --------------		
323	At the last time you became pregnant, did you want to become pregnant at that time or wanted to wait and have pregnancy later or never wanted to have any more children?	1 = Wanted at that time<br>2 = Wanted later<br>3 = Never wanted any more children		
324	Have you ever had a stillbirth or a miscarriage or a menstrual regulation (MR), or an abortion?	1 = MR/D&C/Abortion<br>2 = Miscarriage<br>3 = Stillbirth<br>9 = None		

Section-4: MNCH FP and Nutrition related Knowledge question:

No.	Question	Responses	Code	Direction
**Maternal Health Issues:** [In this part, I would like to ask you some knowledge questions regarding pregnancy and postpartum care, family planning services and nutrition]				
401	Does a pregnant mother require any antenatal check-ups	1 = Yes<br>2 = No		If No. Skip-404
402	Can you please tell me, when the first antenatal check-up is needed actually?	Within first ----------months of pregnancy<br>Do not know----99		
403	Can you please tell me, how many times should a pregnant mother go for antenatal check-ups?	Time (s)-----------------<br>Do not know-----99		
404	What should a woman do about her care during her pregnancy period?<br><br>[Probe for multiple answer]	1 = Eat at least one fist more at every meal<br>2 = Have nutritious food<br>3 = Drink more water and liquids<br>4 = Take iodized salt<br>5 = Keep sound mind and be happy<br>6 = Take additional rest<br>7 = Do not do heavy works<br>8 = Listen to elderly members in the family<br>9 = Other (specify-------------)		
405	Where should a woman have her delivery?	1 = At home<br>2 = At public hospital (Specify-----------------------)<br>3 = At private hospital (For profit)<br>4 = At private hospital (Not- for profit)<br>5 = Other place (Pls. specify------------------------)		
406	If at home, who should conduct the delivery?	01 = Untrained TBA<br>02 = Relative<br>03 = Trained TBA<br>04 = CSBA (GOB)<br>05 = CSBA (Private)<br>06 = SACMO/MA<br>07 = Nurse/Midwife/FWV (GOB)<br>08 = Nurse/Midwife/ Paramedics (Private)<br>09 = Qualified doctor (MBBS)<br>10 = Pharmacist/drug seller/Village doctor<br>11 = NGO health worker (specify--------------)<br>12 = No one<br>13 = Other (specify -------------------------------)<br>99 = Do not know		
407	What are the danger signs of pregnancy that need medical treatment?<br>[Probe for multiple answer]	01 = Hemorrhage/ bleeding<br>02 = Convulsion<br>03 = Severe Headache<br>04 = High fever<br>05 = Blurring of vision<br>06 = Swollen legs/hands/face (edema)<br>07 = High blood pressure<br>08 = Reduced/absent fetal movements<br>09 = Foul smelling discharge from vagina<br>10 = Severe anaemia<br>11 = Rupture of membren before 37 weeks (PROM)<br>12 = Others<br>99 = Do not know		**ANC complication**

No.	Question	Responses	Code	Direction
408	What are the complications in a woman during delivery that needs medical treatment? [Probe for multiple answer]	01 = Excessive bleeding /Hemorrhage<br>02 = Labor lasting more than 12 hours (Prolonged labor)<br>03 = Any body part other than head coming first during delivery (Hand/Leg prolapse/ Breech presentation)<br>04 = Foul smelling discharge from vagina<br>05 = High fever<br>06 = Placenta not delivered within 30 minutes after delivery of the baby (Retained placenta)<br>07 = Convulsion<br>08 = Tetanus<br>09 = Severe abdominal pain<br>10 = Severe chest pain and rapid breathing<br>11 = Other (Specify)---------------<br>99 = Do not know		Delivery complications
409	What are the complications in a woman within 42 days of delivery that needs medical treatment? [Probe for multiple answer]	01 = Excessive bleeding /Hemorrhage<br>02 = Convulsion/ fits<br>03 = High fever<br>04 = Foul smelling discharge from vagina<br>05 = Wound infections<br>06 = Inverted nipples<br>07 = Engorged breast<br>08 = Tetanus<br>09 = Severe lower abdominal pain<br>10 = Severe chest pain and rapid breathing<br>11 = Uterine prolapse<br>12 = Other (Specify)---------------<br>99 = Do not know		PNC complications
**Family Planning Issues**				
410	In your opinion, what is the ideal age of marriage for a male and a female?	a. Male (age in years)<br>b. Female (age in years)		If no opinion or don't know code '99
411	In your opinion, what is the ideal age for a man and a woman to have their first child?	a. Male (age in years)<br>b. Female (age in years)		If no opinion or don't know code '99
412	In your opinion, what is the ideal space of time to wait to have another baby after giving a birth?	In months------------<br>Not sure/ don't know -99		
413	If a married girl gets pregnant in her teenage years (13-19), what are the potential problems she might have to face?	1 = No problem<br>2 = Health risk of mother<br>3 = Health risk of baby<br>4 = Education might get stopped<br>5 = Might not continue job<br>6 = Other (specify ------------------------------------)		
414	There are ways or methods that a couple can use to delay or avoid pregnancy. These are known as family planning (FP) methods. Have you heard about any FP methods?	1 = Yes<br>2 = No		If no, skip to 423
415	Which methods have you heard about?	01= Female sterilization<br>02 = Male sterilization<br>03 = Pill<br>04 = Intrauterine devices (IUD)<br>05 = Injectables<br>06 = Implant/Norplant<br>07 = Condom<br>08 = Safe period<br>09 = Withdrawl<br>10 = Other (specify ----------------------------------)		
416	In general do you approve or disapprove of using Family Planning?	1 = Approve (Continue and don't ask q 418)<br>2 = Disapprove → skip to 418<br>3 = No opinion → skip to 419		

No.	Question	Responses	Code	Direction
417	Why do you approve Family Planning?<br>[Probe for multiple answer]	1 = Economic reasons<br>2 = For health of mother<br>3 = For health of children<br>4 = Difficult to take care of children<br>5 = Other (specify)<br>9 = Not applicable		
418	Why do you disapprove Family Planning?<br>[Probe for multiple answer]	1 = Against religion<br>2 = Fear of side effects<br>3 = Fear of failure of method<br>4 = More children support family<br>5 = Others (specify-----------------------------------)		
419	Do you know about long acting family planning methods?	1 = Yes<br>2 = No		If no, skip to 421
420	If yes, what are the methods that you know?<br>[Probe for multiple answer]	1 = Intrauterine devices (IUD)<br>2 = Implant/Norplant<br>3 = Injectables<br>4 = Other (specify -----------------------------------)		
421	Have you ever been in a position when you needed any FP methods but couldn't get it?	1 = Yes<br>2 = No		If no, skip to 423
422	If it happened, then please tell us why you couldn't get the method that you wanted?			
**Neonatal and Child Health Issues**				
423	Could you name a few basic cares that can be provided to baby after delivery? [Probe for multiple answer]	01 = Drying and wrapping with clean and warm cloth<br>02 = Start breast Feeding /colostrum within 1 hour of delivery<br>03 = Cut the cord with clean blade<br>04 = Eye Care<br>05 = Keep the cord of the baby dry/ cord care<br>06 = Give the newborn a bath at least after 72 hours<br>07 = Avoid shaving during the first month<br>08 = Take Care low birth weight/ pre term baby<br>09 = Refer if any complication observed<br>10 = Other (specify) --------------------------------<br>99 = Do not know		
424	What is to be given to a newborn baby just after birth?	1 = Colostrums (breast milk)<br>2 = Only fresh/plain water<br>3 = Mishri/Sugar water<br>4 = Bottled milk<br>5 = Honey<br>6 = Mustard oil<br>7 = Others (Specify) ________<br>9 = Can't say		
425	How long after birth should a baby be given colostrum (breast milk)?	1 = Within half an hour<br>2 = Within one hour<br>3 = Within 24 hours<br>4 = Second day<br>5 = Others (Specify) ________<br>6 = Later<br>9 = Can't say		
426	Up to which month should a mother feed her baby only breast milk?	--------------Months<br>Don't know-------------99		
427	From which month should a mother feed her baby other supplementary foods besides breast milk?	--------------Months<br>Don't know-------------99		

No.	Question	Responses	Code	Direction
428	What are the danger signs for a newborn baby?<br>[Probe for multiple answer]	01 = Weak cry<br>02 = Absent cry<br>03 = Unable to suck or breast feed<br>04 = Lethargy<br>05 = Cold hands and feet<br>06 = Rapid breathing<br>07 = Chest indrawing<br>08 = High temparature (fever)<br>09 = Convulsion/unconsciousness<br>10 = Umbilical discharge<br>11 = More than 10 skin pustules<br>12 = Red eyes with discharge<br>13 = Jaundice for more than 14 days<br>14 = Low temparature<br>15 = Other -------------------------------<br>99 = Do not know		
429	Does a newborn baby require any vaccinations after birth?	1 = Yes<br>2 = No		
430	What are the vaccines have to be given to a baby?<br>[Probe for multiple answer]	1 = BCG<br>2 = Polio<br>3 = DPT<br>4 = Measles<br>5 = HepB<br>6 = Hib<br>7 = Pentavalent<br>8 = Others (specify)------------<br>9 = Don't know		
431	What are the symptoms of pneumonia?<br>[Probe for multiple answer]	1 = Rapid breathing<br>2 = Chest indrawing<br>3 = Respiratory problem<br>4 = Fever<br>5 = Losing appetite to eat and drink<br>6 = Feeling drowsy all the time<br>7 = Others Specify ___________________<br>9 = Can't say		
**Nutrition**				
432	Have you ever heard of malnutrition among mother and children?	1 = Yes<br>2 = No		If no, skip to q.501
433	From where did you learn about malnutrition among mother and children?<br>[Probe for multiple answer]	01 = Newspaper/magazine<br>02 = Radio<br>03 = TV<br>04 = Billboards<br>05 = Brocheures<br>06 = Poster/leaflet<br>07 = Health workers (NGO/Govt)<br>08 = Family members, neighbors, colleagues<br>09 = Religious leaders<br>10 = Teacher<br>11 = Others (specify-----------)		
434	In your opinion, how serious a problem is malnutrition of mother and child?	1 = Very serious<br>2 = Not Very serious<br>3 = Somewhat serious<br>4 = Don't know		

No.	Question	Responses	Code	Direction
435	What are the symptoms of malnutrition among pregnant and lactating women?<br><br>[Probe for multiple answer]	01 = Weakness/lethargy<br>02 = Repeated infection (e.g., oral thrush, chronic Diarrhea)<br>03 = Thinness<br>04 = Low weight<br>05 = Low height<br>06 = Slow wound healing<br>07 = Difficulty in breathing<br>08 = Anemia<br>09 = Night blindness<br>10 = Goiter<br>11 = Others (Specify:______________<br>99 = Do not know		
436	What are the causes of malnutrition among pregnant and lactating woman?<br><br>[Probe for multiple answer]	01 = Not eating enough food<br>02 = Not eating the right food<br>03 = Diseases<br>04 = Frequent child bearing<br>05 = Inadequate maternal care practices<br>06 = Inadequate access to health services<br>07 = Poor water and sanitation<br>08 = Inadequate knowledge about nutrition<br>09 = Lack of education<br>10 = Poverty<br>11 = Cultural beliefs and practices<br>12 = Others (Specify:--------------<br>99 = Do not know		
437	What are the maternal consequences of malnutrition during pregnancy and lactation?<br><br>[Probe for multiple answer]	1 = Reduced physical capacity (e.g., weakness)<br>2 = Reduced mental capacity (e.g., poor attention)<br>3 = Frequent infections/diseases<br>4 = Reduced household income<br>5 = Maternal mortality<br>6 = Obstructed labor<br>7 = Prolonged labor<br>8 = Others (Specify:-------)<br>9 = Don't know		
438	What are the fetal and child consequences of malnutrition during pregnancy and lactation?<br><br>[Probe for multiple answer]	01 = Miscarriage<br>02 = Stillbirth<br>03 = Intrauterine growth retardation<br>04 = Prematurity<br>05 = Low birth weight<br>06 = Congenital anomalies<br>07 = Impaired growth<br>08 = Impaired motor development<br>09 = Impaired cognitive development<br>10 = Frequent infections<br>11 = Neonatal/Infant mortality<br>12 = Others Specify:________________<br>99 = Don't know		

No.	Question	Responses	Code	Direction
439	What can be done to prevent malnutrition during pregnancy and lactation?<br><br>[Probe for multiple answer]	01 = Increase dietary intake<br>02 = Reduce workload<br>03 = Take iron and folic acid<br>04 = Take multiple micronutrient<br>05 = Take Vitamin A during lactation<br>06 = Improve knowledge on nutrition<br>07 = Treat infections/diseases<br>08 = Ensure appropriate birth spacing<br>09 = Increase access to food<br>10 = Appropriate maternal care practices<br>11 = Adequate access to health services<br>12 = Ensure safe water and sanitation<br>13 = Avoid harmful cultural practices<br>14 = Others (Specify:______________<br>99 = Don't know		

Section-5: MNCH FP and Nutrition related practice question:

No.	Question	Responses	Code	Direction
**Maternal Health (**Now I would like to talk with you about your last pregnancy)				
Antenatal care:				
501	Were you registered or listed at the time of your pregnancy by any healthcare provider or organization?	1= Yes<br>2= No		If no, skip to q.503
502	If so, by whom you were registered?	01 = Untrained TBA<br>02 = Relative<br>03 = Trained TBA<br>04 = CSBA (GOB)<br>05 = CSBA (Private)<br>06 = SACMO/MA<br>07 = Nurse/Midwife/FWV (GOB)<br>08 = Nurse/Midwife/ Paramedics (Private)<br>09 = Qualified doctor (MBBS)<br>10 = Pharmacist/drug seller/Village doctor<br>11 = NGO health worker (specify--------------)<br>12 = Other (specify -------------------------------)<br>99 = Do not know		'FIVDB'a NGO working in this area
503	Did any one discuss with you/your family about birth planning/birth preparedness for your recent delivery?	1= Yes<br>2= No		Pls. Explain birth planning (follow the instruction guideline) [if no skip to 505]
504	If so, who discussed with you about birth planning/ birth preparedness?	01 = Untrained TBA<br>02 = Relative<br>03 = Trained TBA<br>04 = CSBA (GOB)<br>05 = CSBA (Private)<br>06 = SACMO/MA<br>07 = Nurse/Midwife/FWV (GOB)<br>08 = Nurse/Midwife/ Paramedics (Private)<br>09 = Qualified doctor (MBBS)<br>10 = Pharmacist/drug seller/Village doctor<br>11 = NGO health worker (specify--------------)<br>12 = Other (specify -------------------------------)<br>99 = Do not know		

505	What preparation did you take in your last pregnancy?<br><br>[Probe for multiple answer]			1 = Decided about place of delivery<br>2 = Saved money<br>3 = Prepared emergency transport<br>4 = Decided about birth attendant<br>5 = Selected about blood donor<br>6 = Selected referral facilities if any complications<br>7 = Accompanying person during complications to the facility<br>8 = Collected safe delivery kit<br>9 = Others (---------------------)			
506	Did you receive any antenatal check up during your last pregnancy?			1 = Yes<br>2 = No			IF yes, then ask the following question in the table other wise skip to q.511
507	How many times did you have check-ups/ antenatal care during your last pregnancy?			Number of times---------------<br>Do not know/ Can't remember--------99			
No.	Antenatal check-ups	*Place	**Provider	Distance from home	Mode of transport	Cost of services	Direction
507a	ANC-1						Use the given code for place and provder
507b	ANC-2						
507c	ANC-3						
507d	ANC-4						
507e	ANC-5						
507f	ANC-6						
507g	ANC-7						
* place<br>01 = At home<br>02 = Community Clinic<br>03 = Rural Dispencery/ Union Sub-centre<br>04 = Family Welfare Center (FWC)<br>05 = Health & Family Welfare Center (H&FWC)<br>06 = Upazilla Health and Family Welfare Centre (UH&FWC)<br>07 = Maternal and Child Welfare Centre (MCWC)<br>08 = District Hospital (DH)<br>09 = Medical College Hospital<br>10 = NGO health center(Not for profit) [specify------]<br>11 = Private clinic<br>12 = Doctor's private Chamber<br>13 = Others (specify--------------------------)				** Provider<br>01 = Untrained TBA<br>02 = Relative<br>03 = Trained TBA<br>04 = CSBA (GOB)<br>05 = CSBA (Private)<br>06 = SACMO/MA<br>07 = Nurse/Midwife/FWV (GOB)<br>08 = Nurse/Midwife/ Paramedics (Private)<br>09 = Qualified doctor (MBBS)<br>10 = Pharmacist/drug seller/Village doctor<br>11 = NGO health worker (specify--------------)<br>12 = Other (specify -------------------------------)<br>99 = Do not know			
508	Who mainly decided you to attend these providers for ANC visits?			1= You alone<br>2= Husband alone<br>3= You and your husband combinely<br>4= Father in law<br>5= Mother in law<br>6= Mother<br>7= Father<br>8= Brother in law/Sister in law<br>9 = Others (specify--------------)			
509	How many months pregnant were you when you first received antenatal care?			-----------months<br>Do not know------------99			
510	How many months pregnant were you when you last received antenatal care?			-----------months<br>Do not know------------99			

No.	Question	Responses	Code	Direction
511	Sometimes complications may arise during pregnancy. What kind of complications did you experience in your last pregnancy?<br>[Probe for multiple answer]	01 = Hemorrhage/bleeding<br>02 = Convulsion<br>03 = Severe Headache<br>04 = Severe anaemia<br>05 = Blurring of vision<br>06 = Swollen legs/ hands/ face (edema)<br>07 = High blood pressure<br>08 = High fever<br>09 = Foul smelling discharge from vagina<br>10 = Reduced/ absent fetal movements<br>11 = Others<br>99 = No complications		**ANC complications**<br><br>If no, complication skip to q.524
512	In your opinion, which was the most serious complication?	01 = Hemorrhage/bleeding<br>02 = Convulsion<br>03 = Severe Headache<br>04 = Severe anaemia<br>05 = Blurring of vision<br>06 = Swollen legs/ hands/ face (edema)<br>07 = High blood pressure<br>08 = High fever<br>09 = Foul smelling discharge from vagina<br>10 = Reduced/ absent fetal movements<br>11 = Others		
513	Did you seek any care or treatment for that complication?	1 = Yes<br>2 = No		If no, skip to q.524
514	Where did you first seek care/ treatment for these/ this complication?<br>***Record only one answer- where woman or family first sought care/treatment.***	01 = At home<br>02 = Community Clinic<br>03 = Rural Dispencery/ Union Sub-centre<br>04 = Family Welfare Center (H&FWC)<br>05 = Health & Family Welfare Center (H&FWC)<br>06 = Upazilla Health and Family Welfare Centre (UH&FWC)<br>07 = Maternal and Child Welfare Centre (MCWC)<br>08 = District Hospital (DH)<br>09 = Medical College Hospital<br>10 = NGO health center(Not for profit) [specify------]<br>11 = Private clinic<br>12 = Doctor's private Chamber<br>13 = Others (specify--------------------------)		
515	Who provided this care first?	01 = Untrained TBA<br>02 = Relative<br>03 = Trained TBA<br>04 = CSBA (GOB)<br>05 = CSBA (Private)<br>06 = SACMO/MA<br>07 = Nurse/Midwife/FWV (GOB)<br>08 = Nurse/Midwife/ Paramedics (Private)<br>09 = Qualified doctor (MBBS)<br>10 = Pharmacist/drug seller/Village doctor<br>11 = NGO health worker (specify--------------)<br>12 = Other (specify -------------------------------)<br>99 = Do not know		

No.	Question	Responses	Code	Direction
516	Who advised/encouraged you to attend this provider for antenatal complication?	1= You alone<br>2= Husband alone<br>3= You and your husband combinely<br>4= Father in law<br>5= Mother in law<br>6= Mother<br>7= Father<br>8= Brother in law/Sister in law<br>9 = Others (specify--------------)		
517	Why this provider was chosen?	1 = Closer to home<br>2 = Less costly<br>3 = Good behaviour<br>4 = Better service quality<br>5 = Well known to family<br>6 = Others (------------------)		
518	What was the total cost to avail that service?			
519	How far the treatment place was from your home?	-----------------------(Km.)		
520	What was the mode of transport to reach that treatment place?	1 = Rickshaw/Van<br>2 = Boat<br>3 = Tampoo/CNG<br>4 = Micro bus/ Ambulance/Taxi<br>5 = Bus/Train<br>6 = Others (--------------------------------)		
521	Did you get any kind of support from other than your household members to get that service?	1 = Yes<br>2 = No		IF no, Skip to q.424
522	If so, who provided you that support?	1 = Relative<br>2 = Neighbour/Friend<br>3 = Community group (specify---------------)<br>4 = Health Facility<br>5 = Micro-credit organization<br>6 = NGO (specify-----------------------)<br>7 = Others (Specify------------------------)		
523	What kind of support you received that time?	1 = Money<br>2 = Transportation<br>3 = Accompany<br>4 = Demand Side Financing (DSF)<br>5 = Others (specify----------------)		
**Delivery care**				
524	Now I would like to ask you questions about the delivery of your last pregnancy. Where did you give birth to your child?	1 = Own home<br>2 = Parent's home<br>3 = In-laws home<br>4 = Govt. Hospital (specify---------------)<br>5 = Private clinic<br>6 = NGO health center (specify---------------)<br>7 = Other (specify)------		

No.	Question	Responses	Code	Direction
525	Who conducted the delivery?	01 = Untrained TBA<br>02 = Relative<br>03 = Trained TBA<br>04 = CSBA (GOB)<br>05 = CSBA (Private)<br>06 = SACMO/MA<br>07 = Nurse/Midwife/FWV (GOB)<br>08 = Nurse/Midwife/ Paramedics (Private)<br>09 = Qualified doctor (MBBS)<br>10 = Pharmacist/drug seller/Village doctor<br>11 = NGO health worker (specify--------------)<br>12 = No one/Self<br>13 = Other (specify -------------------------------)<br>99 = Do not know		
526	Who advised/encouraged you to attend this provider for delivery care?	1= You alone<br>2= Husband alone<br>3= You and your husband combinely<br>4= Father in law<br>5= Mother in law<br>6= Mother<br>7= Father<br>8= Brother in law/Sister in law<br>9 = Others (specify--------------)		
527	Did you get any kind of support from other than your household members during your delivey?	1 = Yes<br>2 = No		If no. skip to q.530
528	If so, who provided you that support?	1 = Relative<br>2 = Neighbour/Friend<br>3 = Community group (specify---------------)<br>4 = Health Facility<br>5 = Micro-credit organization<br>6 = NGO (specify-----------------------)<br>7 = Others (Specify------------------------)		
529	What kind of support you received that time?	1 = Money<br>2 = Transportation<br>3 = Accompany<br>4 = Demand Side Financing (DSF)<br>5 = Others (specify----------------)		
530	Around the time of the birth of (Name), what kind of complications did you experience?<br>[Probe for multiple answer]	1 = Long labor, that is, regular contraction that lasted more than 12 hours?<br>2 = Excessive bleeding that was so much that you feared it was life threatening?<br>3 = A high fever with bad smelling vaginal discharge?<br>4 = Convulsion?<br>5 = Baby's hands or feet came first during delivery (Prolapse)?<br>6 = Retained placenta (Placenta not delivered within 30 minutes after delivery)<br>7 = Other (specify) ------------------<br>9 = No complications **(skip to q.544)**		IF no complication, then skip to q.544
531	In your opinion, which was the most serious complication?	1 = Long labor, that is, regular contraction that lasted more than 12 hours?<br>2 = Excessive bleeding that was so much that you feared it was life threatening?<br>3 = A high fever with bad smelling vaginal discharge?<br>4 = Convulsion?<br>5 = Baby's hands or feet came first during delivery (Prolapse)?<br>6 = Retained placenta (Placenta not delivered within 30 minutes after delivery)<br>7 = Other (specify) ------------------		
532	Did you seek any assistance for this complication?	1 = Yes<br>2 = No		IF no, then skip to q.544

No.	Question	Responses	Code	Direction
533	If yes, Whom did you see first?	01 = Untrained TBA<br>02 = Relative<br>03 = Trained TBA<br>04 = CSBA (GOB)<br>05 = CSBA (Private)<br>06 = SACMO/MA<br>07 = Nurse/Midwife/FWV (GOB)<br>08 = Nurse/Midwife/ Paramedics (Private)<br>09 = Qualified doctor (MBBS)<br>10 = Pharmacist/drug seller/Village doctor<br>11 = NGO health worker (specify--------------)<br>12 = Other (specify -------------------------------)<br>99 = Do not know		
534	Who advised/motivated you to attend this provider for delivery complication?	1= You alone<br>2= Husband alone<br>3= You and your husband combinely<br>4= Father in law<br>5= Mother in law<br>6= Mother<br>7= Father<br>8= Brother in law/Sister in law<br>9 = Others (specify--------------)		
535	Why this provider was chosen?	1 = Closer to home<br>2 = Less costly<br>3 = Good behaviour<br>4 = Better service quality<br>5 = Well known to family<br>6 = Others (------------------)		
536	What was the total cost to avail that service?			
537	How far the treatment place was from your home?	-----------------------(Km.)		
538	What was the mode of transport to reach that treatment place?	1 = Rickshaw/Van<br>2 = Boat<br>3 = Tampoo/CNG<br>4 = Micro bus/ Ambulance/Taxi<br>5 = Bus/Train<br>6 = Others (--------------------------------)		
539	Did you get any kind of support from other than your household members to get that service?	1 = Yes<br>2 = No		If No, skip to q.542
540	If so, who provided you that support?	1 = Relative<br>2 = Neighbour/Friend<br>3 = Community group (specify---------------)<br>4 = Health Facility<br>5 = Micro-credit organization<br>6 = NGO (specify-----------------------)<br>7 = Others (Specify------------------------)		
541	What kind of support you received that time?	1 = Money<br>2 = Transportation<br>3 = Accompany<br>4 = Demand Side Financing (DSF)<br>5 = Others (specify----------------)		
542	Were you referred by him/her?	1 = Yes<br>2 = No		IF no, then skip to q.544
543	If you were referred, then where did you go?	1 = Pharmacy<br>2 = NGO health center (specify) -----------<br>3 = Private clinic<br>4 = Govt. Hospital/ clinic<br>5 = Private Chamber<br>6 = Other (specify) ---------------------------		

No.	Question	Responses	Code	Direction
Postnatal care				
544	Now I would like to ask you some questions about your postpartum care.<br>After (NAME) was born, did you have a medical checkup?	1 = Yes<br>2 = No		**PNC**<br>IF no, then skip to q.548
545	How long after delivery did the first check up take place?	a. -----------hours after delivery<br>b. -----------days after delivery<br>c. -----------weeks after delivery<br>Don't know-----------999		
546	How many checkups did you have?	-------------- times		
547	Who checked you first time after delivery? (Provider for 1st PNC check-up)	01 = Untrained TBA<br>02 = Relative<br>03 = Trained TBA<br>04 = CSBA (GOB)<br>05 = CSBA (Private)<br>06 = SACMO/MA<br>07 = Nurse/Midwife/FWV (GOB)<br>08 = Nurse/Midwife/ Paramedics (Private)<br>09 = Qualified doctor (MBBS)<br>10 = Pharmacist/drug seller/Village doctor<br>11 = NGO health worker (specify--------------)<br>12 = Other (specify -------------------------------)<br>99 = Do not know		
548	What are the complications did you experience within 42 days of your last delivery?<br>[Probe for multiple answer]	01 = Severe/excessive bleeding<br>02 = Foul smelling discharge from vagina<br>03 = High fever<br>04 = Convulsion<br>05 = Inverted nipples<br>06 = Tetanus<br>07 = Severe abdominal pain<br>08 = Engorged breast<br>09 = None<br>10 = Other (specify) -------------		IF no complication then code 09 and skip to q.562
549	In your opinion, which was the most serious complication?	01 = Severe/excessive bleeding<br>02 = Foul smelling discharge from vagina<br>03 = High fever<br>04 = Convulsion<br>05 = Inverted nipples<br>06 = Tetanus<br>07 = Severe abdominal pain<br>08 = Engorged breast<br>09 = None<br>10 = Other (specify) -------------		
550	Did you seek any care or treatment for any of these/this complication(s)?	1 = Yes<br>2 = No		IF no. skip to q.562

No.	Question	Responses	Code	Direction
551	If yes, Whom did you see first?	01 = Untrained TBA<br>02 = Relative<br>03 = Trained TBA<br>04 = CSBA (GOB)<br>05 = CSBA (Private)<br>06 = SACMO/MA<br>07 = Nurse/Midwife/FWV (GOB)<br>08 = Nurse/Midwife/ Paramedics (Private)<br>09 = Qualified doctor (MBBS)<br>10 = Pharmacist/drug seller/Village doctor<br>11 = NGO health worker (specify--------------)<br>12 = No one<br>13 = Other (specify -------------------------------)<br>99 = Do not know		
552	Who decided you to attend this provider for postnatal complication?	1= You alone<br>2= Husband alone<br>3= You and your husband combinely<br>4= Father in law<br>5= Mother in law<br>6= Mother<br>7= Father<br>8= Brother in law/Sister in law<br>9 = Others (specify--------------)		
553	Why this provider was chosen?	1 = Closer to home<br>2 = Less costly<br>3 = Good behaviour<br>4 = Better service quality<br>5 = Well known to family<br>6 = Others (------------------)		
554	What was the total cost to avail that service?			
555	How far the treatment place was from your home?	-----------------------(Km.)		
556	What was the mode of transport to reach that treatment place?	1 = Rickshaw/Van<br>2 = Boat<br>3 = Tampoo/CNG<br>4 = Micro bus/ Ambulance/Taxi<br>5 = Bus/Train<br>6 = Others (--------------------------------)		
557	Did you get any kind of support from other than your household members during to get that service?	1 = Yes<br>2 = No		If no, skip to q.560
558	If so, who provided you that support?	1 = Relative<br>2 = Neighbour/Friend<br>3 = Community group (specify---------------)<br>4 = Health Facility<br>5 = Micro-credit organization<br>6 = NGO (specify-----------------------)<br>7 = Others (Specify-----------------------)		
559	What kind of support you received that time?	1 = Money<br>2 = Transportation<br>3 = Accompany<br>4 = Demand Side Financing (DSF)<br>5 = Others (specify----------------)		
560	Were you referred by him/her?	1 = Yes<br>2 = No		IF no. skip to q.562

No.	Question	Responses	Code	Direction
561	If you were referred, then where did you go?	1 = Pharmacy<br>2 = NGO health center (specify) -----------<br>3 = Private clinic<br>4 = Govt. Hospital/ clinic<br>5 = Private Chamber<br>6 = Other (specify) ---------------------------		
**Newborn care**<br>Now, I would like to ask you some specific questions about what was done with (Name) immediately following delivery				
562	What was used to cut the cord?<br>(In case of home delivery)	1 = Blade from delivery bag<br>2 = Blade from other sources<br>3 = Bamboo strips<br>4 = Scissors<br>5= Other (specify)-------------------------------------<br>6 = Cord was not cut (Skip to q.564)<br>9 = Do not know/ Not applicable (Skip to q.564)		
563	Was the ----- (instrument) boiled before the cord was cut?	1 = Yes<br>2 = No<br>9 = Do not know		
564	How long after (Name) was born was the body wiped?	Minutes: ☐☐<br>Not wiped---------98<br>Do not know------99		
565	How long after (Name) was born was the body wrapped?	Minutes: ☐☐<br>Not wrapped---------98<br>Do not know------99		
566	How long after delivery was (Name) bathed for the first time?<br><br>IF LESS THAN ONE DAY, RECORD IN HOURS<br>IF LESS THAN ONE WEEK RECORD IN DAYS	1 = Hours: ☐☐<br><br>2 = Days: ☐☐<br><br>3 = Weeks: ☐☐<br><br>Not Bathed----------998<br>Do not know--------999		
567	Did you ever breastfeed (Name)?	1 = Yes<br>2 = No		If no, skip to q. 570
568	How long after birth did you first put (Name) to the breast?<br><br>IF LESS THAN 1 HOUR, RECORD ‘00’ HOURS<br>IF LESS THAN 24 HOURS, RECORD HOURS.<br>OTHERWISE, RECORD DAYS	Immediately ---000<br><br>1 = Hours: ☐☐<br><br>2 = Days: ☐☐		
569	Was (Name) given colostrum immediately after his/her birth?	1= Yes<br>2 = No		
570	In the first three days after delivery, was (Name) given anything to drink other than breast milk?	1= Yes<br>2 = No		If no, skip to q. 572
571	What was (Name) given to drink?<br><br>RECORD ALL LIQUIDS MENTIONED	01 = Milk (Other than breast milk)<br>02 = Plain water<br>03 = Sugar or glucose water<br>04 = Gripe water<br>05 = Sugar-salt-water solution<br>06 = Fruit juice<br>07 = Infant formula<br>08 = Tea/Infusions<br>09 = Honey<br>10 = Other (specify)------------------------	☐☐<br>☐☐<br>☐☐<br>☐☐	

No.	Question	Responses	Code	Direction
572	When did you shave hair of (Name) for the first time?	1 = Just after birth<br>2 = < 24 hours of birth<br>3 = Second day<br>4 = Third day to one month<br>5 = One month later<br>6 = Don't remember<br>7 = Yet to shave		
573	During the first month (28 days) of life did (Name) have any problems?<br>[Probe for multiple answer]	01 = Weak cry<br>02 = Absent cry<br>03 = Unable to suck or breast feed<br>04 = Lethargy<br>05 = Cold hands and feet<br>06 = Rapid breathing<br>07 = Chest indrawing<br>08 = High fever<br>09 = Convulsion/unconsciousness<br>10 = Umbilical discharge<br>11 = More than 10 skin pustules<br>12 = Red eyes with discharge<br>13 = Jaundice for more than 14 days<br>14 = None of the above<br>15 = Other-------------------------		If answer is None then code 14 and skip to q.579
574	Did you seek any care or treatment for any of these/this complication(s)?	1 = Yes<br>2 = No		If no, skip to q.579
575	If yes, Whom did you see first? Any one else?	01 = Untrained TBA<br>02 = Relative<br>03 = Trained TBA<br>04 = CSBA (GOB)<br>05 = CSBA (Private)<br>06 = SACMO/MA<br>07 = Nurse/Midwife/FWV (GOB)<br>08 = Nurse/Midwife/ Paramedics (Private)<br>09 = Qualified doctor (MBBS)<br>10 = Pharmacist/drug seller/Village doctor<br>11 = NGO health worker (specify--------------)<br>12 = Other (specify ------------------------------)<br>99 = Do not know		
576	Were you referred by him/her?	1 = Yes<br>2 = No		If no, skip to q.579
577	If you were referred, then where did you refer?	1 = Pharmacy<br>2 = NGO health center (specify) -----------<br>3 = Private clinic<br>4 = Govt. Hospital/ clinic<br>5 = Private Chamber<br>6 = Other (specify) ---------------------------		
578	Did you go to that referral place?	1 = Yes<br>2 = No		
**Child Care**				
579	Are you still breastfeeding (Name)?<br>CHECK-CHILD ALIVE?	1 = Yes<br>2 = No<br>3 = Not applicable		
580	For how many months did you exclusively breastfeed (Name)?	Months: ☐☐<br>Do not know-----99		
581	When did you start supplementary food to your baby after birth?	------------ days<br>-------------months<br>Yet to start ----------------88<br>Do not know---------------99		
582	Did (Name) ever receive any vaccination to prevent him/her from getting diseases?	1 = Yes<br>2 = No		If no, skip to q.584

No.	Question	Responses	Code	Direction
583	Please tell me if (Name) received any of the following vaccines:	1 = BCG<br>2 = Polio<br>3 = DPT<br>4 = Measles<br>5 = HepB<br>6 = Hib<br>7 = Pentavalent<br>8 = Others (specify)------------<br>9 = Don't know		
584	Did your baby suffer from fever or cough or cold or difficulty in breathing or pneumonia at any time in last 6 months?	1 = Yes<br>2 = No<br>Do not know------9		If answer is no or do not know, skip to q.587
585	Did you seek advice or treatment for (Name) for the illness from any source?	1 = Yes<br>2 = No		If answer is no, skip to q.587
586	Who has provided your baby this care or treatment?	01 = Untrained TBA<br>02 = Relative<br>03 = Trained TBA<br>04 = CSBA (GOB)<br>05 = CSBA (Private)<br>06 = SACMO/MA<br>07 = Nurse/Midwife/FWV (GOB)<br>08 = Nurse/Midwife/ Paramedics (Private)<br>09 = Qualified doctor (MBBS)<br>10 = Pharmacist/drug seller/Village doctor<br>11 = NGO health worker (specify--------------)<br>12 = No one<br>13 = Other (specify -------------------------------)<br>99 = Do not know		
**Family planning**				
587	If you are not currently pregnant, which family planning methods you or your husband is using right now?	01= Female sterilization<br>02 = Male sterilization<br>03 = Pill<br>04 = IUD<br>05 = Injectables<br>06 = Implant/Norplant<br>07 = Condom<br>08 = Safe period<br>09 = Withdrawl<br>10 = Other (specify ---------------------------------)<br>11 = None (skip to q.601)		If answer is 11, then skip to q.601
588	From where did you receive FP methods/commodity?	01 = Untrained TBA<br>02 = Relative<br>03 = Trained TBA<br>04 = CSBA (GOB)<br>05 = CSBA (Private)<br>06 = SACMO/MA<br>07 = Nurse/Midwife/FWV (GOB)<br>08 = Nurse/Midwife/ Paramedics (Private)<br>09 = Qualified doctor (MBBS)<br>10 = Pharmacist/drug seller/Village doctor<br>11 = NGO health worker (specify--------------)<br>12 = No one<br>13 = Other (specify -------------------------------)<br>99 = Do not know		

Section-6: Healthcare seeking:

No.	Questions	Types of services	Provider	Cost of services	Distance from Household (Km)
601	From where do you or any of your household women seek **general** healthcare primarily?	General health services (e.g., fever, cough, cold, etc)			
602	From where do you or any of your household women seek **maternal** healthcare primarily?	ANC			
603		Delivery			
604		PNC			
605	From where do you or any of your household members seek **child** healthcare primarily?	Newborn/child health services			
606	From where do you or any of your household women seek **family planning services** primarily?	FP services			
607	From where do you or any of your household women seek **nutrition** care primarily?	Nutrition			

**Types of services:**	**Provider list**
General: (e.g., fever, cough, cold, pain, etc)<br>Maternal: (ANC-Antenatal care, delivery care, Postnatal care)<br>Child: Newborn care; under-5 child care<br>Family planning: FP commodity; consultation, etc<br>Nutrition: Consultation; treatment; suupliment; growth monitoring; messages	01 = Untrained TBA; 02 = Relative; 03 = Trained TBA;<br>04 = Community health worker (specify)-<br>05 = CSBA (GOB------------); 06 = CSBA (Private----------)<br>07 = SACMO/MA (GOB); 08 = Nurse/Midwife (GOB); 09 = Nurse/Midwife (NGO/Private); 10 = Qualified doctor (MBBS); 11 = Pharmacist/ drug seller/Village doctor; 12 = Homeopath; 13 = Traditional healer; 14= Other (specify)—

Section-7: Expenditure for recent delivery and willingness to pay:

No	Question	Responses	Code	Direction
701	What was the transport cost (up and down)	---------------------------- Tk.		Write '00' if there in no such expenditure or not applicable
702	**Cost of Delivery at home/hospital**			
	a. Admission fees	……………………….Tk		
	b. Laboratory test fees	……………………….Tk		
	c. Medicines	……………………….Tk		
	d. Hospital charges (Bed/cabin charge)	……………………….Tk		
	e. Consultation fees	……………………….Tk		
	f. Other expenditure (Specify----------------)	……………………….Tk		
	g. Total	……………………….Tk		
703	**Cost incurred for other purposes**	……………………….Tk		
	a. Tips	……………………….Tk		
	b. Sweets	……………………….Tk		
	c. Broker	……………………….Tk		
	d. Gift	……………………….Tk		
	e. Others----------------	……………………….Tk		
	f. Total	……………………….Tk		
704	What was the total cost for the newborn care?	……………………….Tk		
705	What price do you/your household currently pay for normal delivery at home?	--------------------------tk.		WTP questions
706	Would you pay a medium or increase if we can ensure private CSBA for normal delivery?	1= Yes<br>2 = No		If no, skip to q.708
707	If yes, would you pay a higher increase?	1= Yes<br>2 = No		
708	If no, would you pay a lower increase?	1= Yes<br>2 = No		
709	What is the highest price you would pay?	----------------------Tk.		
710	What would you do if the price is too high?	------------------		

Section-8: Mass media exposure, cell phone use and NGO/micro-credit involvement

No.	Question	Responses	Code	Direction
801	Do you read newspaper or magazine?	1= Yes<br>2 = No		If no, skip to 803
802	How often do you read newspaper or magazine?	1 = Almost every day<br>2 = At least once a week<br>3 = Less than once a week		
803	Do you listen to the radio?	1= Yes<br>2 = No		If no, skip to 805
804	How often do you listen to the radio?	1 = Almost every day<br>2 = At least once a week<br>3 = Less than once a week		
805	Do you watch television?	1= Yes<br>2 = No		If no, skip to 807
806	How often do you watch television?	1 = Almost every day<br>2 = At least once a week<br>3 = Less than once a week		
807	Are you involved with any micre-credit or community based organization (CBO) organization?	1= Yes<br>2 = No		If no, skip to 809
808	What are the organizations that you are involved?	1 = Grameen Bnak<br>2 = BRAC<br>3 = BRDB<br>4 = ASHA<br>5 = Proshika<br>6 = CBO (----------)<br>7 = Other (specify---)		CBO such as Village Development Committee, Clubs etc
809	Do they provide any kind of health related support (e.g., direct Health Services, support for referral, loan, informatiom, etc) in your community?	1 = Yes<br>2 = No		
**Utility of cell phone use in healthcare seeking**				
810	Do you own any mobile phone?	1 = Yes<br>2 = No		
811	Does your family own a mobile phone in your household?	1 = Yes<br>2 = No		
812	Do you have 24 hours access to a mobile phone?	1 = Yes<br>2 = No		
813	Have you ever communicated with anyone for healethcare purpose?	1 = Yes<br>2 = No		IF no skip to q. 901
814	If yes, with whom you usaully communicate?	1 = Healthcare provider (specify----------)<br>2 = Relative<br>3 = Community support group members<br>4 = Others (specify-----------------------)		
815	For what purposes did you communicate?	1 = Pregnancy complication<br>2 = Delivery complication<br>3 = Postnatal complication<br>4 = Neonatal sickness<br>5 = Child's sickness<br>6 = Other (specify---------------------------)		
816	Did they respond it?	1 = Yes<br>2 = No		IF no skip to q. 901
817	Do you think that the phone call was helpful in that situation?	1 = Yes<br>2 = No		

Section-9: Community group's activity related to MNCH, FP and Nutrition in study area:

No.	Question	Responses	Code	Direction
901	Do you know any of community groups working in your area related to maternal and child health, family planning and Nutrition to motivate community people?	1 = Yes<br>2 = No<br>Do not know ------------9		IF answer is 'no or don't know' then skip to q. 904
902	What are the community groups working in this area?	a.-------------<br>b.--------------<br>c.----------------		
903	What are the services or support they usually provide?	1 = Communication<br>2 = Transportation<br>3 = Fund<br>4 = Others (----------)		
904	Did any group meeting on Maternal and child health, family planning and Nutrition take place in or around your area in the last six months?	1 = Yes<br>2 = No		
905	Did you take part in any group meeting on health or family planning in the last six months?	1 = Yes<br>2 = No		

Section-10: Child mortality

1001	Has any under -5 child of your family died in the last five years?	1 = Yes<br>2 = No			IF answer is 'no then complete the interview
1002	If yes, how many children have died?				
1003	How older was the baby (s) when s/he died and what were causes of death?	**Age**	**Cause of death**		
		a.Year--------Months------------Day	aa.		
		b. Year-------Months------------Day	bb.		
**Thank the respondent for her time and complete the interview**					

Ending time of data collection: ☐☐ ☐☐ **Date:** ☐☐ ☐☐

How much time was needed to complete the interview: ☐☐ ☐☐

For Official Use only

A	Name of the area			
B	Result Status of the interview	Complete--------------------------------1<br>Incomplete after 3 attempts-----------------2<br>Refusal---3		
C	Data collector's name and Signature----------Date: -------/------/20_			
D	Supervisor's name and Signature-------------Date: -------/------/20_			
E	Research investigator's name and Signature--Date: -------/------/20_			
F	Data entry person's name and Signature------Date: -------/------/20_			
