# Supplementary figures and images for: Filling the human resource gap through public-private partnership: Can private, community-based skilled birth attendants improve maternal health service utilization and health outcomes in a remote region of Bangladesh?
Source: PLoS One. 2020 Jan 17;15(1):e0226923. doi: 10.1371/journal.pone.0226923 (PMC6968857; doi:10.1371/journal.pone.0226923)

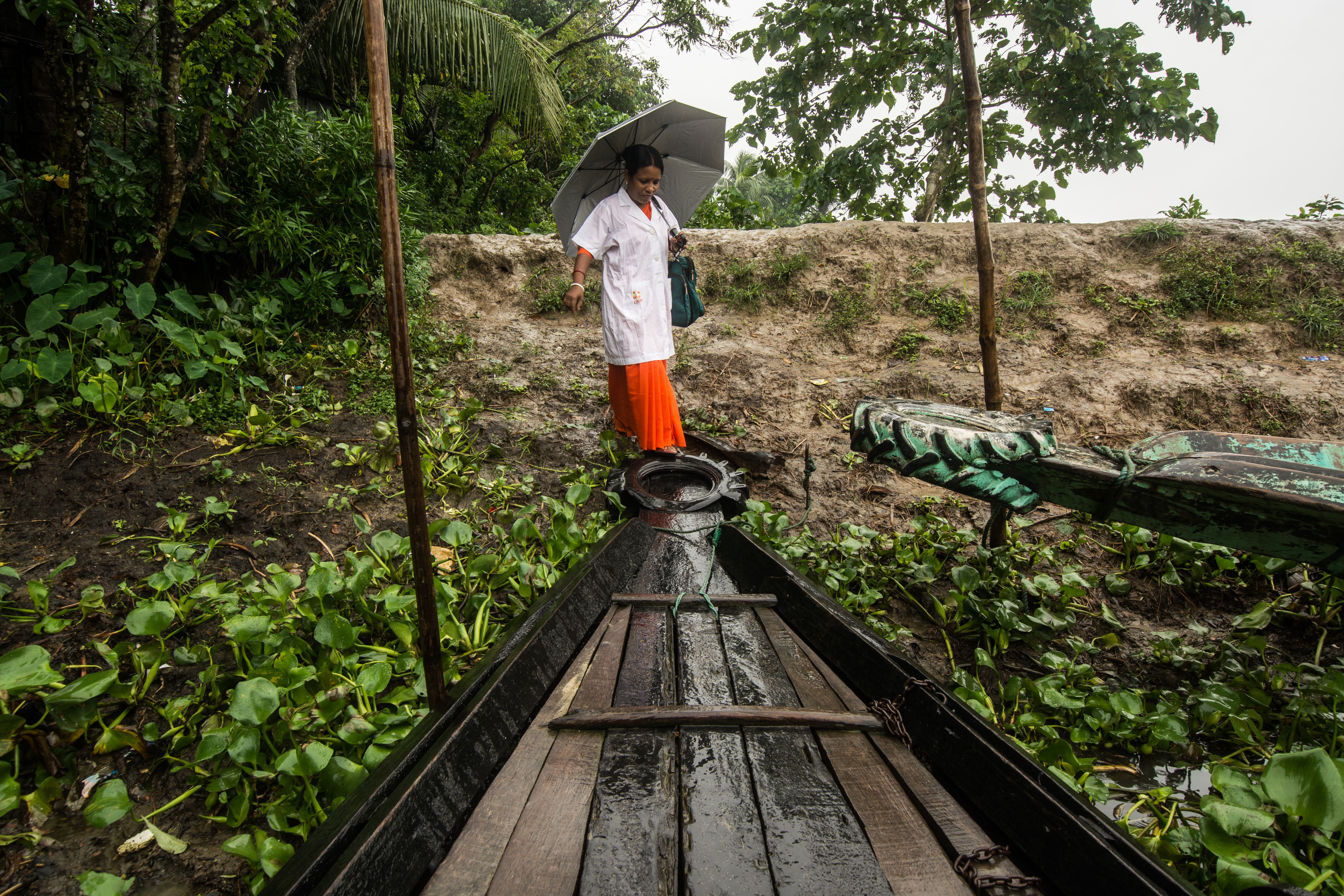

Supplement: S3 File — Shirpa was trained as a P-CSBA and now provides health services to her small village and surrounding community. Because Shirpa’s village is surrounded by water she must take a boat every morning to make her household visits. (JPG) [file pone.0226923.s003.jpg]
